# Advances in Chemical Constituents, Clinical Applications, Pharmacology, Pharmacokinetics and Toxicology of *Erigeron breviscapus*

**DOI:** 10.3389/fphar.2021.656335

**Published:** 2021-09-02

**Authors:** Ruixia Wu, Yan Liang, Min Xu, Ke Fu, Yangliu Zhang, Lei Wu, Zhang Wang

**Affiliations:** ^1^ College of Pharmacy, Chengdu University of Traditional Chinese Medicine, Sichuan, China; ^2^ College of Ethnomedicine, Chengdu University of Traditional Chinese Medicine, Sichuan, China

**Keywords:** Erigeron breviscapus, breviscapine, scutellarin, clinical application, pharmacology

## Abstract

Dengzhanxixin (DZXX), the dried whole plant of *Erigeron breviscapus* (Vaniot) Hand.-Mazz., belonging to *Compositae* and first published in *Materia Medica of South Yunnan* by Lan Mao in the Ming Dynasty (1368 AD–1644 AD), is included in Medicinal Materials and Decoction Pieces of the 2020 edition of the Pharmacopeia of the *People’s Republic of China*. Its main chemical components are flavonoids that mainly include flavonoid, flavonols, dihydroflavones, flavonol glycosides, flavonoid glycosides, coffee acyl compounds, and other substances, such as volatile oil compounds, coumarins, aromatic acids, pentacyclic terpenoids, phytosterols, and xanthones. Among them, scutellarin and 1,5-dicoffeoylquininic acid are the main active components of DZXX. DZXX has pharmacological effects, such as improving cerebral and cerebrovascular ischemia, increasing blood flow, inhibiting platelet aggregation, promoting antithrombotic formation, improving microcirculation, reducing blood viscosity, protecting optic nerves, exhibiting anti-inflammatory properties, scavenging free radicals, and eliciting antioxidant activities. It is widely used in the treatment of cardiovascular and cerebrovascular ischemic diseases, kidney diseases, liver diseases, diabetic complications, and glaucoma. Pharmacokinetic studies have shown that the active components of DZXX have a low bioavailability and a high elimination rate in vivo. Nevertheless, its utilization can be improved through liposome preparation and combination with other drugs. Acute and subacute toxicity studies have shown that DZXX is a safe medicinal material widely used in clinical settings. However, its target and drug action mechanism are unclear because of the complexity of its composition. In this paper, the clinical application and pharmacological toxicology of DZXX are reviewed to provide a reference for further studying its active components and action mechanism.

## Introduction

Dengzhanxixin (DZXX), the dried grass of *Erigeron breviscapus* (Vaniot) Hand.-Mazz. belonging to *Compositae*, is a perennial herb, 5–50 cm tall, with woody rhizomes and thick rhizomes. The stem is upright, with a few branches in the middle, and the whole plant is covered with multicellular short bristles or mixed with glandular hairs. The leaves are mainly concentrated at the base and are in the shape of a rosette. The leaves are obovate-lanceolate or wide-spoon-shaped. The base is half embracing, and the upper part is often reduced to a small bracteole with no petiole. The flower head is solitary at the top of the stem or branch, and the involucre is hemispherical. DZXX is mainly distributed in Yunnan, Guangxi, Guizhou, and other places. It is a characteristic medicinal material of Yunnan, and is listed as the four major cardio-cerebral vascular medicines along with ginkgo, panax notoginseng and salvia. DZXX is commonly used in Miao nationality, Yi, Tibetan, Dai, and other ethnic groups and was first published in the Ming Dynasty Lanmao’s *Materia Medica of South Yunnan* (about the 14th to 15th century). It has been used to treat ischemic cerebrovascular diseases, such as paralysis; it is included in the “Medicinal Materials and Decoction Pieces” of the 2020 edition of the *Pharmacy of the People’s Republic of China*. In the 1930s, the whole herb of the Yunnan Miao herb DZXX was used to treat stroke and hemiplegia. In the 1960s, under the impetus of the Chinese medicine movement, Luo San of Yunnan Miao Medicine cut the whole plant during the flowering period of DZXX at an altitude of 1,000–1,400 m, mixed with eggs and water and steamed for 15 min before taking it. The effect of treating cerebral palsy is remarkable. Since it was introduced to the world, it has been extensively applied and studied. It has officially entered the ranks of national legal medicinal materials, and a series of unilateral Chinese medicine preparations has been developed, used as raw materials, and applied in clinical practice. In 1994, DZXX was listed as a high-technology product of the National Torch Program and became an essential Chinese medicine for emergency treatment in Chinese medicine hospitals across the country. In 2000, it was listed as a national protected variety of Chinese medicine and prescription drug (Yang, 2014). Such drugs are administered to treat diseases such as hemiplegia, coronary heart disease, cerebral thrombosis, rheumatism, and microcirculation disorders, and they have a wide range of treatment, a definite curative effect, and minimal side effects. Especially for the treatment of cardiovascular and cerebrovascular diseases and their sequelae, the curative effect is more obvious, the therapeutic effect is more than 95% (Tao, 2016).

Since the inclusion of the “Yunnan Pharmaceutical Standards” in 1997, according to incomplete statistics, the annual purchase of DZXX, which is widely used as a raw material in the pharmaceutical industry in Yunnan Province, has reached more than 1 million kg, mainly from wild DZXX resources. The total amounts of privately collected wild resources were 300, 400, 1,000, 1,100, 1,200, and 1,000 tons in 1995, 1996, 1997, 1998, 1999, and 2000, respectively. The drug product based on the total flavonoids of DZXX requires about 1.5 million kg of raw materials. For breviscapine tablets, breviscapine injection, and other patented medicines based on breviscapine, the demand for raw materials is about 1.2 million kg. DZXX injection produced on the basis of phenolic acid compounds, such as scutellarin and total caffeic acid esters, requires about 400,000 kg of raw materials for medicinal materials, and the total demand is about 3.1 million kg. The domestic market demand for DZXX continues to increase by 15–20% per year. At present, the annual amount of wild DZXX available in Yunnan Province does not exceed 500,000 kg. With several years of collection, wild DZXX resources have become scarce (Zhang et al., 2013; Tao, 2016). At present, the wild resources of DZXX have been exhausted, and artificial cultivation of DZXX has become the only way to maintain the sustainable development of the medicine industry of DZXX. The survey shows that the stock of DZXX resources from Yunnan Province is only about 800–1,000 tons, and the supply can only meet 18.5% of the market demand (Wang N. et al., 2012). Now Yunnan has mastered the mature technology of planting breviscapine, and the planting scale of breviscapine in the province has reached 10,000 Mu (6.67 square kilometers). The output of medicinal materials reached 4,000 tons, from the serious gap in market demand to the resource demand that can guarantee the development of the breviscapine industry. At the same time, in view of the current artificial cultivation of mixed sources, poor stability, difficulty in seedling breeding, low seed production efficiency, lag in the development of high-quality and high-yield standardized planting technology, and serious pests and diseases, Yunnan Province has established a technical system for seed production and floating seedlings of DZXX and cultivate a professional planting technical team. Promote large-scale, standardized and industrialized production of *Erigeron breviscapus.*

The *2017 China Dengzhanhua Industry Development Report* shows that in 2015, the sales of cardio-cerebrovascular Chinese patent medicines made from DZXX in Yunnan accounted for 1.71% of the national public medical institution cardio-cerebrovascular Chinese patent medicine market, compared with other categories such as Danhong injection, there is still a large market space. According to investigations and studies, the average treatment course of breviscapine-related preparations used in hospitals is 7–18 days. Most of them are used in elderly patients, mainly for patients with cerebral infarction and coronary heart disease and angina pectoris. At the same time, the medication indications and instructions are in line with the high rate. The dosages are all within the scope of the instructions, and the incidence of adverse events is less than 1%. A few adverse reactions occur mostly within half an hour. Most of them are immediate and mild, mainly middle-aged and elderly people, mainly manifested as skin allergies and headaches., Abdominal pain, the adverse reaction may be related to the use of super-indications and combined medication (Gao C. et al., 2007; Gao Z.et al., 2008; Ji et al., 2009; Li YY. et al., 2015; Xiang et al., 2018).

DZXX has become a common drug in the clinical treatment of cardiovascular and cerebrovascular related diseases. With the deepening of system, organ, and molecular levels, the scope of its clinical applications is expanding. This paper searches the published literature, using DZXX as key word; the search scope includes CNKI, Wanfang Database, Web of science database, Springer Link foreign language journal database and other databases. In this paper, the clinical application and pharmacological toxicology of DZXX and its related preparations are reviewed to clarify the main treatment-related mechanism of DZXX and its preparation and to provide ideas for further research.

## Chemical Composition and Preparation of DZXX

**FIGURE 1:**
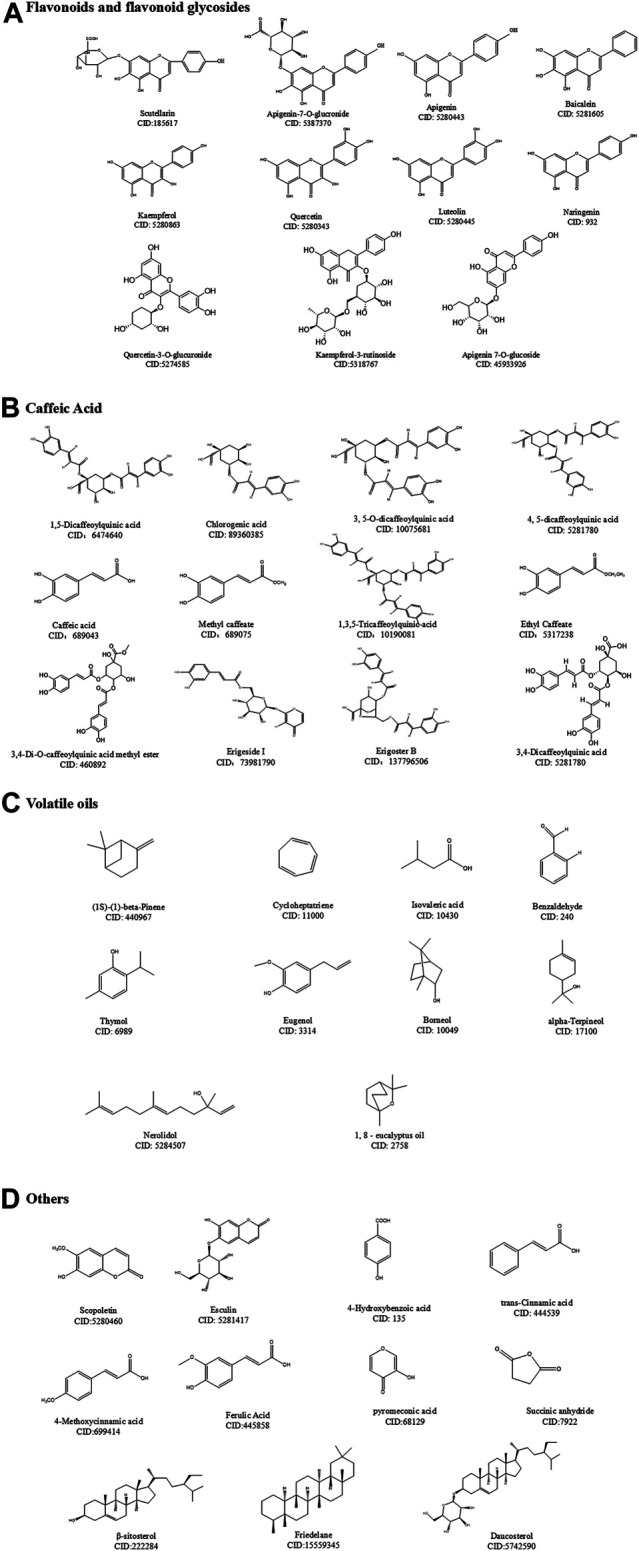
Some typical chemical structures in four types of DZXX.

At present, 25 kinds of flavonoids, 46 kinds of caffeoyl compounds, 78 kinds of volatile oils, and nearly 40 kinds of other compounds, including coumarin, pentacyclic triterpenes, aromatic acids, phytosterols, and oxythracrone, are isolated from DZXX (Zhang et al., 2000a; Zhang et al., 2000b; Guo et al., 2019). Table 1 and Figure 1 below show some familiar structural formulas. The chemical structure painted by *ChemDraw* Software.

**TABLE 1:** Some typical chemical components in four types of DZXX.

Classify	No.	Compound name	Molecular formula
Flavonoids and flavonoid glycosides	1	Scutellarin	C_21_H_18_O_12_
	2	Apigenin-7-O-glucronide	C_22_H_20_O_12_
	3	Apigenin	C_15_H_10_O_5_
	4	Baicalein	C_15_H_10_O_5_
	5	Naringenin	C_15_H_12_O_5_
	6	Kaempferol	C_15_H_12_O_6_
	7	Quercetin	C_15_H_12_O_7_
	8	Luteolin	C_15_H_10_O_6_
	9	Apigenin 7-O-glucoside	C_21_H_20_O_10_
	10	Quercetin-3-O-glucuronide	C_21_H_18_O_13_
	11	Kaempferol-3-rutinoside	C_27_H_30_O_15_
Caffeic acid	12	1,5-Dicaffeoylquinic acid	C_25_H_24_O_12_
	13	Chlorogenic acid	C_16_H_18_O9
	14	3,5-O-dicaffeoylquinic acid	C_25_H_24_O_12_
	15	4, 5-dicaffeoylquinic acid	C_25_H_24_O_12_
	16	Caffeic acid	C9H8O4
	17	methyl caffeate	C_25_H_24_O_12_
	18	Ethyl caffeine	C_11_H_12_O4
	19	1,3,5-Tricaffeoylquinic acid	C_34_H_30_O_15_
	20	3,4-Di-O-caffeoylquinic acid methyl ester	C_26_H_26_O_12_
	21	Erigeside I	C_20_H_20_O_11_
	22	3,4-Dicaffeoylquinic acid	C_25_H_24_O_12_
	23	Erigoster B	C_26_H_24_O_13_
Volatile oils	24	(1 S)-(1)-beta-Pinene	C_10_H_16_
	25	Cycloheptatriene	C_7_H_8_
	26	Isovaleric acid	C_5_H_10_O_2_
	27	Benzaldehyde	C_7_H_6_O
	28	Thymol	C_10_H_14_O
	29	Eugenol	C_10_H_12_O_2_
	30	Borneol	C_10_H_18_O
	31	α-terpineol	C_10_H_18_O
	32	Nerolidol	C_15_H_26_O
	33	1, 8 - eucalyptus oil	C_10_H_18_O
Others	34	Scopoletin	C_10_H8O_4_
	35	Esculin	C_15_H_16_O_9_
	36	4-Hydroxybenzoic acid	C_7_H_6_O_3_
	37	Trans-Cinnamic acid	C_9_H_8_O_2_
	38	4-Methoxycinnamic acid	C_10_H_10_O_3_
	39	Ferulic Acid	C_10_H_10_O_4_
	40	Pyromeconic acid	C_5_H_4_O_3_
	41	Succinic anhydride	C_4_H_4_O_3_
	42	β-sitosterol	C_29_H_50_O
	43	Friedelane	C_30_H_52_
	44	Daucosterol	C_35_H_60_O_6_

### Flavonoids and Flavonoid Glycosides

Flavonoids and their glycosides are the main components of DZXX, which are mainly composed of flavonoids, flavonols, dihydroflavonoids, flavonol glycosides and flavonoid glycosides. Among them, scutellarin is considered to be the main active ingredient in flavonoids, and it is also the most studied ingredient in DZXX (Guo et al., 2019). See the Table 1 and Figure 1A below for details.

### Caffeic Acid

DZXX caffeoyl compounds mostly exist in the form of a combination of the nucleus and different numbers of caffeic acid, in the form of quinic acid (CQA), 2,7-anhydro-3-deoxy-2-octylpyrrolidone acid (CDOA), 2, 7-anhydro-2-octylpyrrolidone acid (COA), 1- (2'-γ-pyrone) four types as main. The caffeoylquinic acid compounds in DZXX are mostly, and the main active ingredient is 1,5-diCQA. Table 1 and Figure 1B below show some of the caffeoyl (Guo et al., 2019). See the Table and Figure below for details.

### Volatile Oils

Volatile oil compounds are mostly long-chain fatty acids, cyclics, long-chain fatty alkanes and other compounds. Table 1 and Figure 1C below show some of the volatile oils (Guo et al., 2019). See the Table and Figure below for details.

### Others

Dzxx also contains coumarins, aromatic acids, pentacyclic triterpenes, phytosterols, xanthones and other compounds. The following Table 1 and Figure 1D below show some of the other compounds (Zhang et al., 2000a; Zhang et al., 2000b; Guo et al., 2019). See the Table and Figure below for details.

DZXX has been developed into numerous formulations, involving injections, granules, tablets, dispersible tablets, capsules, mixtures, extracts, and dripping pills. The number of formulations is nearly 20 products. According to the State Food and Drug Administration (Medical Products Administration, 2020), currently marketed single-prescription DZXX preparations are mainly oral and injectable. Their raw materials include breviscapine (3 approval numbers) and DZXX extract (2 approval numbers), and most of them are preparations with scutellarin as raw materials, oral preparations include breviscapine tablets (DZHSP) (66 approval number), DZXX granules (2 approval number), breviscapine dispersion tablets (4 approval document numbers), breviscapine chewable tablets (1 approval document number), Dengzhanhua dripping pills (1 approval document number), breviscapine dripping pills (2 approval document numbers). Injection preparations are available DZXX injection (DZXXI) (2 approval document numbers), breviscapine injection (DZHSI) (24 approval document numbers), breviscapine for injection (6 approval document numbers), breviscapine sodium chloride injection (1 approval document), breviscapine glucose injection (1 approval number). There are less preparations using DZXX extract as raw materials, mainly oral DZXX mixture (2 approval number), DZXX capsules (4 approval document number), and DZXX soft capsule (1 approval document number).

DZXXI was approved by China Food and Drug Administration in 2005 and listed in the Chinese Pharmacopoeia. DZXX injection is a sterile aqueous solution (injection) made by extracting phenolic components from *E. breviscapus*. The analysis of HPLC charts and ultraviolet spectra shows that the main component of DZXX includes caffeoyl derivatives (or caffeic acid analogs) and contains a small amount of scutellarin and other flavonoids with a pH between 5.5 and 7.5 (Yang et al., 2005). Flavonoids are represented by scutellarin, which is recognized as the main active ingredient of DZXX injection. Phenolic acids include caffeic acid, chlorogenic acid, and dicaffeoylquinic acid series isomers (Zhang et al., 2002; Zhou L. et al., 2011). DZXX injection is prepared by water decoction, alcohol precipitation (80% ethanol concentration), ethyl acetate extraction, water solubility (pH 8–8.5), activated carbon treatment, and liquid preparation (Yang et al., 2005). In addition, the extraction rate of DZXX after pulverization increases by about 0.17 percentage points (a relative increase of about 15%); however, the whole herb is fed more often because of difficulty in filtrating the components and no difference in extracting from the whole herb or cutting into sections. Flavonoid glycosides in DZXX are more polar, and ethyl acetate cannot be used to completely extract flavonoid glycosides. A certain amount of ethanol can be added to adjust the polarity of extracts and improve the extraction rate (Tang et al., 2002). According to the first edition of the Pharmacopoeia of the People’s Republic of China in 2020, the quality standards of scutellarin for flavonoids and 1,3-O-dicaffeoyl quinic acid for total phenolic acids in DZXX injection are established. Each 1 ml contains flavonoids based on scutellarin (C_21_H_18_O_12_) and should be equivalent to 0.40–0.60 mg. The total caffeic acid content per milliliter is calculated as 1,3-O-dicaffeoylquinic acid (C_25_H_24_O_12_), which should be 2.0–3.0 mg.

Breviscapine injection is a sterile aqueous solution made of flavonoid gluconate glycosides extracted from the whole plant of DZXX and extracted flavonoids. The main component of this solution is scutellarin, a small amount of apigenin-7-O-β-D-glucuronide, and other flavonoid glycosides, but it has no caffeoyl derivatives; its pH is 6.3–8.3 (Tian et al., 2014). The drug is included in Volume 20 of the Prescriptions of Traditional Chinese Medicines of the Ministry of Health of the People’s Republic of China. The quality standard of breviscapine injection has been established on the basis of scutellarin. Its content is determined through spectrophotometry. Breviscapine is based on scutellarin, and it should be 95.0–105.0% of the amount of specimens. Studies have found that the pH of breviscapine injection is about 6.8. When Na_2_HPO_4_ is used as a cosolvent, the color, pH, and drug content of the drug solution become more stable. In addition, the combination of EDTA-2Na and NaHSO_3_ as an antioxidant is more conducive to improving the stability of the drug; when the amount of added sodium bisulfite is 0.2%, the color of the injection is light, and its stability is better (Wang et al., 2010).

Experimental studies have found that scutellarin is easily soluble in water, methanol, and ethanol below 90% concentration, slightly soluble in absolute ethanol and acetone, and hardly soluble in ethyl acetate. Most caffeoyl derivatives are easily soluble in water, ethanol, and water. Ethanol, methanol, and acetone are soluble in ethyl acetate but hardly soluble in chloroform. Other DZXX preparations, such as DZXX capsules, Dengzhanhua granules, and Yimaikang tablets, are all water extracts or 60–80% ethanol extracts, so they contain both flavonoids and caffeoyl derivatives (Yang et al., 2005). Some details about DZXX series preparations are presented in Table 2.

**TABLE 2:** Information of DZXX series preparations.

Name of preparation	Dosage Forms	Drug standards	Number of approved symbols (i)	Preparation process	Content determination	Main treatment	Chemical constituents	Active ingredients and content
Dengzhanxixin Injection (DZXXI)	Injection	People’s Republic of China Pharmacopoeia 2020 Edition	2	Dzxx and water decoction twice, filter, compress, add water dilution, adjust Ph, filter to get filtrate and precipitate. Precipitation washing, adjustment Ph, drying into dry paste powder; filtrate elution, adjustment Ph, extraction, compression into clear paste. Add injection water, sodium chloride, activated carbon, mix well, filter, seal, sterilize	High performance liquid chromatography (flavone in baicalin) UV-Vis spectrophotometry (determination of total caffeic acid O-1,3-dicaffeoylquinic acid (C25H24012)	Blood stasis block, apoplexy hemiplegia, limb numbness, mouth-eye skew, speech acerbity and chest pain; ischemic stroke, coronary heart disease angina pectoris	1,3- O- dicaffeoyl quinic acid,3,5-di- O- caffeoyl quinic acid,4,5-di- O- caffeoyl quinic acid, 5-caffeoyl quinic acid, chlorogenic acid,4-caffeoyl quinic acid, caffeic acid,1,3-dicaffeoyl quinic acid, wild baicalin, isochlorogenic acid B,3,5- dicaffeoyl quinic acid, breviscapine,4,5-dicaffeoyl quinic acid, cinnamic acid; p-methoxy cinnamic acid (Liu et al., 2017; Chen, 2017)	3-caffeoylquinic acid (53.6 mg/L), caffeic acid (62.4 mg/L),1,5-2-O-caffeoylquinic acid (107 mg/L), baicalin (412 mg/L) (Wang et al., 2018a)
Breviscapine injection (DZHSI)	Injection	Preparation of Traditional Chinese Medicine by Pharmaceutical Standard of the Ministry of Health of the People’s Republic of China Volume 20	24	To dissolve 0.26 g ethylenediamine tetraacetate disodium in 280 ml of water for injection, add breviscapine 1 g, stir suspension, add sodium bicarbonate to dissolve Ph7-7.5, breviscapine, add water for injection to 400 ml, add 0.1% activated carbon of solution volume, adsorb 20 min, to remove carbon fine filter, seal tank after qualified test, after 100°C15 steam sterilization, lamp inspection, packaging	Spectrophotometry (breviscapine in breviscapine)	Sequelae of stroke, coronary heart disease, angina	Scutellarin, Breviscapine	Scutellarin (4. 05 mg/ml), Breviscapine (Zhao and Gu, 2011; Zheng and Li, 2011)
Dengzhanxixin Granules (DZXXG)	Granules	People’s Republic of China Pharmacopoeia 2020 Edition	2	Take erigeron breviscapus, grind, heat reflux extraction, combine the extraction solution, filter, filtrate to thick paste, add sucrose and dextrin appropriate amount, mix, make into granules, dry;Or add lactose, dextrin right amount, mix, make grain, dry	High performance liquid chromatography (DZXX by Scutellarin (C21H18O12)	Cerebral collaterals obstruction, stroke hemiplegia, heart obstruction, chest arthralgia; ischemic stroke, coronary heart disease angina pectoris see the above syndrome	——	——
Breviscapine tablets (DZHSP)	tablet	People’s Republic of China Pharmacopoeia 2020 Edition	66	Take breviscapine 20 g, add starch 68 g, dextrin 60 g, sift, mix well, make granules, dry, press into 1,000 tablets, that is	High performance liquid chromatography (breviscapine in scutellarin (C21H18O12)	Sequelae of stroke, coronary heart disease, angina	scutellarin,Apigenin-7- O- glucuronide	scutellarin
Breviscapine drop pill (DZHSDP)	Pill	——	2	The polyethylene glycol-6000 was heated and melted, then stearic acid and poloxamer 188 were added, then the breviscapine powder was added after complete melting, and the mixture was transferred to the burette under the condition of heat preservation. The molten mixtureps the molten mixture into liquid paraffin in ice water bath, condenses and solidifies into pellets, washing off liquid paraffin	——	Stroke sequelae, coronary heart disease, angina pectoris	scutellarin	scutellarin (12.1,12.8,12.4 mg/g) (Gui Zhouby, 2009)
Dengzhanhua Pill (DZHP)	Pill	——	1	Take the Asarum extract and add the molten matrix while stirring, stir well, drop the coolant, collect the drop pills, and dry	——	Ischemic cerebrovascular disease and cerebral hemorrhage paralysis, fundus retinal vein occlusion, coronary heart disease, vasculitis dermatosis, rheumatism	scutellarin	scutellarin (21,19.3,19.2 mg/g) (He and Liu, 2002)
Dengzhanxixin Capsule (DZXXC)	Capsule	Notice on Transforming Chinese Medicine Trial Standards into Promulgates (2)	4	the breviscapine asarum was chopped and refluxed twice with 60–80% ethanol, filtered, combined with extract, added activated carbon, refluxed, filtered, filtrate concentrated to paste to add starch, vacuum drying, crushing, sifting, coating, loading capsule	Determination of Scutellaria baicalensis by UV spectrophotometry	Blood stasis obstruction of collaterals stroke; chest obstruction; cerebral infarction and coronary heart disease, angina pectoris see the above syndrome	scutellarin, Chomaconic Acid (Long and Huang, 2011)	scutellarin
Dengzhanxixin soft capsule (DZXXSC)	Capsule	——	1	Add suspension aid into diluent, mix well, add erigeron breviscapus extract powder, add antioxidant and preservative, stir and set aside.The prepared liquid is poured into the liquid bucket, and the mold presses the soft capsule	High performance liquid chromatography (scutellarin in breviscapine)	——	Scutellarin, 1,3-O- dicaffeoyl quinine	scutellarin (6.04 mg/grain) (Huang et al., 2013)
Breviscapine for injection (DZHSFI)	Injection	Chinese Medicine Standard of the Ministry of Health of the People’s Republic of China	6	Apply dzxx, add appropriate amount of water for injection, adjust Ph value to 7 with sodium carbonate, stir to dissolve, add appropriate amount of mannitol for injection, remove bacteria, filter, determine content, pack, freeze-dry, seal。	Spectrophotometry (scutellarin in breviscapine)	apoplexy and its sequelae, coronary heart disease, angina pectoris	Scutellarin (Lin et al., 2009)	scutellarin
Breviscapine Glucose Injection (DZHSGI)	Injection	——	1	reigerine was mixed with PEg-400 and water for injection, followed by the addition of diglycine peptide and the adjustment of Ph with sodium hydroxide to 6.5–8.5. Glucose was added after dissolution, followed by filtration. The concentrated solution was determined by constant volume, ultrafiltration, filling, nitrogen filling, film adding, plug adding, and high temperature sterilization	——	Cerebral infarction, sequelae of cerebral hemorrhage; coronary heart disease, angina pectoris, myocardial infarction and hyperviscosity; other ischemic and microcirculation disorders	Breviscapine, scutellarin, Glucose	scutellarin
Breviscapine Sodium Chloride (DZHSSC)	Injection	——	1	Disodium ethylenediamine tetraacetic acid and sodium chloride were weighed and dissolved in water for injection, then brevosin was added for stirring, l-arginine 1 was added for stirring and dissolving, sodium bisulfite was added for stirring, mixed well, water for injection, nitrogen was filled in cans, pressure plug was pressed, high temperature sterilization was obtained	High performance liquid chromatography (scutellarin in breviscapine)	Stroke, cerebral thrombosis, cerebral hemorrhage and its sequela; coronary heart disease, angina pectoris; refractory cervical syndrome, vertebrobasilar insufficiency, microcirculation disorders	scutellarin, Sodium chloride	——
Breviscapine ChewableTablets (DZHSCT)	Tablet	——	1	Breviscapine, mannitol, lactose, stevioside, sift and mix well, make granules with 2% povidone K30, dry, spray orange essence, add magnesium stearate, press tablets	——	Sequelae of stroke, coronary heart disease, angina。	scutellarin	——
Breviscapine Dispersible Tablets (DZHSDT)	Tablet	——	4	The mixture of breviscapine, microcrystalline cellulose MCC, crosslinked carboxymethyl cellulose sodium CMS-Na, crosslinked polyvinylpyrrolidone dextrin, etc. was mixed evenly with water as wetting agent to prepare soft material, sifting, granulation, drying at 60°C, whole grain, adding magnesium stearate, mixing, pressing tablets	High performance liquid chromatography (scutellarin in breviscapine)	Stroke sequelae, coronary heart disease, angina pectoris	Breviscapine, scutellarin, celery, high baicalein (Liang et al., 2012)	scutellarin
Dengzhanxixin Mixture (DZXXM)	Mixture	——	2	Using 70% ethanol to extract breviscapine,8,6,6 times ethanol for 3 times each time 1.5 h. After the extract is concentrated, the impurity is removed, the excipient is added, the glass bottle is filled, sterilized, and the lamp is sterilized. Inspection and other processes are made	UV-spectrophotometry (total flavonoids in rutin) High performance liquid chromatography (scutellarin in breviscapine)	Hemiplegia, wind dampness pain, coronary heart disease angina pectoris, fundus retinal vein obstruction, vascular inflammatory skin disease	Chlorogenic acid, caffeic acid,1,3- O- dicaffeoyl quinic acid, scutellarin (Mei et al., 2013)	scutellarin (1.18 mg/ml)Cai et al. (2014)

## Clinical Applications

### Cerebrovascular Diseases

#### Cerebral Ischemia

Li divided 84 patients with ischemic stroke into a control group and an observation group. The control group was treated with modern medical treatment and given citicoline, aspirin, low-molecular dextran, and nimodipine. The observation group was treated with DZXX injection. After 60 days, DZXX injection improved the patient’s blood lipid index and blood viscosity, and significantly improved the prognosis of stroke patients. (Wang and Guo, 2011; Li, 2018). Yang Nan divided 120 patients with initial ischemic stroke and recurrent ischemic stroke into a control group and a breviscapine injection treatment group. Their results reveal that DZXX injection can effectively reduce plasma C-reactive protein and inhibit its pro-inflammatory effects, indicating that breviscapine can promote the stability of cerebral atherosclerotic plaques through anti-inflammatory activities and improve the prognosis of stroke (Yang, 2009). In another clinical study, breviscapine injection reduced plasma alpha granule membrane protein (GMP-140), platelet activating factor (PAF) and platelet aggregation rate in patients with cerebral ischemia, and improved transient ischemic attack (TIA) patients with platelet activity index, the total effective rate of treatment reached 94.34%, and the total incidence of adverse events was lower than that of the conventional treatment group (Jiang, 2017). A large number of clinical data indicate that DZXX preparations can improve patients’ cerebral ischemia and improve neuromotor function by anti-inflammatory, anti-thrombotic, improving blood rheology, and protecting nerves. The detailed study of DZXX and its related preparations in the treatment of patients with cerebral ischemia is shown in Table 3.

**TABLE 3:** Clinical application of DZXX preparations in various diseases.

Disease	Sample size			Gender	Age/(year)	Medication		Treatment time	Effects	References
	T	C	Total	M/F	T/C	Treatment group(T)	Control group(C)			
Cerebral ischemia	42	42	84	T:21/21 C:22/20	T:54–85 C:53–84	DZXXI, iv	Cytocholine, Aspirin, Low Molecular Dextran, Nimodipine	60d	Treatmet group reduce the blood lipid, improved the score of limb motor function, the effective rate (92.9%) was significantly higher than the control group (76.2%)	Li, (2018)
	60	60	120	T:31/29 C:34/26	T:52–79 C:49–78	DZXXI 20 ml,iv	Routine thrombolysis, anticoagulation and antiplatelet therapy	15d	Treatmet group reduced C-reactive protein	Yang et al. (2009a)
	40	40	80	T:24/16 C:27/13	T:47–68 C:48–70	DZXXI 40 ml,iv	Clopidogrel bisulfate tablet,75 mg, oral	14d	Treatmet group reduce the whole blood viscosity, plasma viscosity, fibrinogen and platelet aggregation rate and the levels of TG, TC and LDL-C	Wang, (2015a)
	305	102	407	——	——	DZXXI 40 ml, iv + Aspirin100 mg	0.9%sodium chloride injection 20 ml, iv +aspirin 100 mg	14d	The curative effect of treatment group was significantly higher than that of control group	Yu et al. (2008)
	30	30	60	T:19/11 C:18/12	T:48–73 C:47–71	DZXXI 40 ml,iv	Routine anticoagulation, thrombolysis and cerebral circulation improvement therapy	14d	Treatmet group reduce the levels of NSE and S100β	Fang et al. (2013)
	30	30	60	T:21/9 C:20/10	T:62.84 ± 7.3 C:63.9 ± 8.4	DZXXI 20–40 ml,iv	nimodipine 30 mg,oral	30d	Treatmet group reduce the serum hs-CRP and serum uric acid UA levels	Jin and Luo, (2013)
	53	53	106	T:32/21 C:30/23	T:42–76 C:44–75	DZHSI 40 mg,iv	Routine oxygen inhalation, anti-infection therapy + aspirin 100 mg+ low molecular weight heparin calcium 5000U subcutaneous injection	14d	Treatmet group reduce the serum GMP-140, PAF and platelet aggregation rate, and the total effective rate of 94.34% was significantly higher than that of 79.25% in the control group	Jiang, (2017)
	40	40	80	T:26/14 C:24/16	T:42–75 C:41–74	DZHSI 20 mg,iv	tetramethylpyrazine 80 mg,iv	15d	In the treatment group, the neurological function, hemodynamic indexes and blood lipid were significantly improved, and the total effective rate was 86.6%, and the total effective rate was 76.8% in the control group	Yao et al. (2014)
Cerebral infarction	42	45	87	T:26/16 C:27/18	T:60 ± 6.12 C:61 ± 7.15	DZXXI 40 ml,iv+Mannitol lowers blood pressure, decreases blood sugar	Mannitol antihypertensive, hypoglycemia + compound salvia miltiorrhiza injection 16 ml,iv	14d	The levels of circulating endothelial cells (CEC), plasma endothelin (ET) and calcitonin gene-related peptide (CGRP) were significantly decreased in the treatment group	Wu et al. (2006)
	43	43	86	T:30/13 C:29/14	T:60.03 ± 13.15 C:59.75 ± 12.47	DZXXI 20–40 ml,iv	Routine antihypertensive and antiplatelet aggregation therapy	14d	Serum levels of Hcy, HsCRP, TNF-α and IL-1 were lower than those in the control group, and serum levels of HIF-1α and Caspase-3 and serum uric acid were significantly lower than those in the control group after treatment	Yin et al. (2017)
	50	50	100	T:24/26 C:27/23	T:64.12 ± 5.47 C:64.56 ± 7.34	DZXXI 30 ml,iv	Xueshuantong Injection 0.3 g,iv	15d	Plasma t-PA activity increased, while PAI-1 activity decreased	Wu et al. (2011)
	18	21	39	——	T:46–79 C:45–80	DZXXI 30 ml,iv	Routine blood pressure lowering and antiplatelet therapy	14d	Plasma PaO2 and PaCO2 were significantly improved in the treatment group	Lin, (2012)
	28	28	56	T:18/10 C:19/9	T:43–75 C:45–75	DZXXI 40 ml,iv	Routine dehydration, anti-platelet aggregation, nourishing brain cells	14d	The expression of S100βprotein and the degree of neurological impairment were significantly decreased in the treatment group	Zhen et al. (2011)
	43	43	86	——	——	DZXXI 40 ml,iv	Butylphthalide and Sodium Chloride Injection 100 ml, Cattle Encephalon Glycoside and Ignotin Injection 10 ml,iv+Aspirin 100 mg+Clopidogrel 75 mg+conventional therapy	14d	The serum levels of endothelin (ET) and nitric oxide (NO) in the treatment group decreased more than those in the control group. The total effective rate of the observation group was 95.3%, and that of the control group was 79.1%	You et al. (2014)
	20	20	40	T:12/8 C:14/6	T:65.3 ± 11.94 C:65.05 ± 9.58	DZXXI 30 ml, iv+ct	Blood pressure control, anti - poly, lipid regulation and other routine treatment	7d	In the treatment group, serum VEGF and EPCs were significantly increased, and serum MMP-9 was inhibited	Hou et al. (2015)
	28	28	56	T:18/10 C:19/9	T:43–75 C:45–75	DZXXI 40 ml,iv	conventional therapy	14d	Serum BDNF expression was significantly increased in the treatment group	Zhen et al. (2012)
	36	33	69	T:21/15 C:20/13	——	DZXXI 40 ml,iv	Piracetam Injection 250 ml,iv+Nimodipine 40 mg, aspirin 100 mg, oral	15d	Serum adhesion molecule sICAM-1 and CD11b/CD18 expression were significantly decreased in the treatment group	Wang et al. (2006a)
	35	33	68	T:22/13 C:21/12	T:53–89 C:55–87	DZXXI 40 ml,iv+Regular treatment	low molecular dextran 500 ml and naofukang injection 250 ml,iv+Nimodipine 40 mg, oral	——	The total effective rate (88.6%) in treatment group was significantly higher than that in control group (66.7%), and the contents of CD62P, IL-6 and TNF-α in platelets were significantly decreased	Wang et al. (2005a)
	40	40	80	T:26/14 C:22/18	T:36–83 C:38–82	DZHSI 50 mg,iv	Danshen injection 16 ml,iv	14d	Neurological function improved obviously in the treatment group	Liu, (2009)
	36	32	68	T:22/14 C:15/17	T:62.8 C:61.5	DZHSI 30 ml,iv	Danshen injection 10 ml,iv	14d	In the treatment group, cognitive function and behavioral ability as well as lipid indexes were significantly improved	Mo and Lu, (2006)
	38	38	76	T:21/17 C:22/16	T:50–81 C:52–80	DZHSI 15 ml,iv	sodium ozagre 80 mg,iv	6w	The clinical treatment, neurological deficit score and hemorheology indexes in the treatment group were significantly better than those in the control group	Xia, (2013)
	58	50	108	T:39/19 C:33/17	T:45–78 C:49–76	DZHSI 40 ml,iv	Routine antihypertensive therapy and supportive therapy	4w	TC, TG and LDL were decreased and HDL was increased in the treatment group, and the total effective rate was 93.1% higher than that of the control group (58.0%)	Feng and Hu, (2005)
Hyperlipemia	34	34	68	T:24/10 C:19/15	T:66.95 ± 9.05 C:66.50 ± 7.68	DZXXI 30 ml,iv	Compound Danshen 20 ml, iv	14d	treatment group decreased the whole blood viscosity, plasma viscosity, fibrinogen, total cholesterol and triacylglycerol, the basic cure rate was 64.71%, which was significantly higher than that of the compound control group (32.35%)	Wu et al. (2004)
	25	—	25	T:15/10	——	DZHSI 25 mg,iv	——	14d	In the treatment group it was significantly reduced TC、TG、LDL-C,MDA,Ox-LDL,ET, evaluated HDL-C,SOD,NO	Yu, (2011)
Hyperviscosity	120	80	200	——	——	DZXXI 40 ml,iv	Compound Danshen injection 20 ml,iv	14d	The whole blood viscosity, plasma viscosity, hematocrit, fibrinogen, cholesterol, triglyceride and high density lipoprotein cholesterol were significantly changed in the treatment group	Chang, (2008)
	68	—	68	T:40/28	T:38–75	DZXXI 30 ml,iv	compound danshen injection 20 ml,iv	14d	The plasma viscosity, platelet adhesion rate and erythrocyte aggregation index were significantly decreased in the treatment group	He and Liu, (2004)
	37	39	76	T:20/17 C:22/17	T:45–83 C:40–80	DZXXI 30 ml,iv	Danshen injection 20 ml,iv	14d	In the treatment group, fibrinogen was significantly decreased and thromboplastin time was prolonged	Shen and Li, (2002)
	48	—	48	T:26/22	——	DZHSI 15 ml,iv	Routine hypotensive, hypoglycemic and symptomatic management	14d	Treatment group significantly reduced whole blood high cut, low cut viscosity, plasma viscosity, fibrinogen	Han et al. (2004)
Cerebrovascular diseases	30	30	60	——	——	DZXXI 30 ml,iv+Regular treatment	compound danshen injection 20 ml,iv	8w	Treatment group reduced platelet CD41, CD63, CD62p and plasma D-dimer	Luo, (2003)
	42	40	82	T:24/18 C:29/11	T:47–76 C:46–78	DZXXI 20 ml,iv+Regular treatment	Conventional treatment + enteric aspirin 100 mg	2w	Treatment group decreased fibrinogen, blood viscosity and TC, TG and LDL-C levels	Zhao et al. (2008)
	40	40	80	T:30/10 C:28/12	T:42–80 C:43–79	DZXXI 20 ml,iv+Regular treatment	Routine enteric aspirin, statins, lipid-lowering β-blockers, and nitrates were given	2w	Treatment group decreased the expression levels of IL-6, CRP and TNF-α	Li and Yang, (2014)
	40	40	80	——	——	DZXXI 40 ml,iv	nitroglycerin 5 mg,iv	——	Treatment group decreased CEC and ET, the total effective rate (93.3%) was significantly higher than that of the control group (72.7%)	Chen et al. (2003)
	40	40	80	——	——	DZXXI 180 mg,iv	Routine antiplatelet and anticoagulant therapy	2w	Treatment group decreased CD63, CD61, GMP-140, GPⅡb/Pa-A,PAC-1 and PAGT	Zhang, (2015a)
	60	60	120	T:36/24 C:34/26	T:55.20 ± 8.20 C:57.80 ± 7.40	DZXXI 40 ml,iv	Routine nitrate beta-blockers, angiotensin-converting enzyme inhibitors, antiplatelets, and anticoagulant statin therapy	2w	The total effective rate of the treatment group was significantly higher than that of the control group	Wang et al. (2012b)
	30	30	60	T:16/14 C:15/15	T:68.7 C:66.1	DZXXI 100 mg,iv	Routine low molecular weight heparin, aspirin, nitrates, statins lipid-regulating drug treatment	——	Treatment group decreased the levels of FG,VWF,t-PA and PAI-1	Zhao et al. (2010)
	30	30	—	——	——	DZHSI 50 mg,iv	Aspirin Enteric-coated Tablets100 mg, Metoprolol tartrate tablet 12.25 mg, Simvastatin tablet 20 mg, Nitroglycerin, oral + low molecular weight heparin calcium 4000 U, subcutaneous injection	14d	The observation group significantly decreased the whole blood high cut viscosity, plasma viscosity, RBC aggregation index, RBC deformability index, COL and ADP induced platelet aggregation rate	Jiang et al. (2018)
Pulmonary disease	45	45	90	T:28/17 C:26/19	T:54–84 C:52–85	DZXXI 20 ml	Anti-infection, oxygen therapy, spasmolysis, antiasthmatic strong heart diuretic, correct acid-base and water, electrolyte imbalance and other comprehensive treatment	21d	The total effective rate of the treatment group was 93.3% higher than that of the control group (68.9%). The treatment group increased PaO2, pH value, decreased PaCO2, plasma D-dimer, Hb	Li et al. (2006)
	104	104	208	T:71/33 C:65/39	T:63–85 C:58–87	DZXXI 20 mg, iv	Routine oxygen inhalation, asthma, phlegm, strong heart, diuresis, dilation of blood vessels	14d	The indexes of whole blood high shear viscosity and fibrinogen were significantly decreased in the treatment group	Cao et al. (2006)
	46	38	74	T:32/14 C:27/11	T:54.23 ± 0.43 C:53.43 ± 0.56	DZXXI 50 ml,iv	Routine treatment: sensitive antibiotics, cough, expectorant, antiasthmatic	20d	The levels of serum ET,sVCAM-1, SICAM-1 and NO were significantly decreased in the treatment group	Kong et al. (2010)
	34	34	68	T:27/5 C:26/5	T:80–95 C:80–93	DZXXI 30 ml,iv	Salmeteroticasone powder inhaler 50 μg/500 μg,inhalation	60d	The pulmonary function indexes, plasma IL-8, TNF-α, CRP levels, blood viscosity, hematocrit and fibrinogen were improved in the treatment group compared with before treatment	Xiao et al. (2015)
	38	38	76	T:20/18 C:19/19	T:21–64 C:22–61	DZHSI 30 mg,iv	Routine anti-infection, antispasmolysis drugs and correction of the patient’s water electrolyte and acid-base disorders and other treatment	15d	The effective rate of reducing the mean pulmonary pressure in the treatment group was 97.01%, and that in the control group was 85.3%	Sun, (2012)
	58	58	116	——	——	DZHSI 40 mg, iv	Routine anti-infection, oxygen inhalation and cough, phlegm, asthma and other routine treatment	14d	The total effective rate of the treatment group was 94.8%, which was significantly higher than that of the control group (81.0%). The forced expiratory volume at the first second (FEV1) and forced expiratory volume at the first second/forced vital capacity (FEV1/FVC) were significantly increased, and CD4^+^ CD4+/CD8+ were significantly higher than that of the control group	Hu et al. (2015)
	28	28	56	——	——	DZHSI 20 mg, iv	Routine anti-infection, spasmolysis, antiasthmatic and other basic treatment	14d	The total effective rate of the treatment group was higher than that of the control group, the proportion of CD3^+^, CD4+T lymphocytes and NK cells, the ratio of CD4+/CD8+ and the level of serum Ig A in peripheral blood were higher than that of the control group	Li and Jia, (2015)
Kidney disease	30	30	60	T:20/10 C:21/9	T:22–69 C:27–69	DZXXI 40 ml,iv	Bailing capsule, oral	15d	The treatment group significantly decreased serum creatinine (SCR) and increased renal effective plasma flow (ERPF), and the effective rate was 86.7% significantly higher than that of the control group (63.3%)	Yin et al. (2010)
	38	——	38	T:23/15	T:60–90	DZXXI 20 ml,iv	——	14d	In the treatment group, BUN, SCR, SBP, DBP, LDL, whole blood viscosity, fibrinogen (FI), whole blood low cut viscosity, cholesterol (CH), triglyceride (TG), maximum platelet aggregation rate decreased, and HDL increased	Cheng et al. (2005)
	50	50	100	T:28/22 C:26/24	T:3–10 C:4–11	DZXXI 10 ml,iv	Captopril tablets,0.3 mg/kg,oral	14d	The BUN, SCR, β2-MG content, IL-8, TNF-α, and IL-6 levels of the treatment group decreased	Liu et al. (2020a)
	40	36	76	T:30/10 C:28/8	T:36–78 C:45–83	DZHSI 20 ml,iv	Routine antihypertensive therapy and supportive therapy	28d	Serum creatinine (CR), blood urea nitrogen (BUN),β2-MG and 24 h urinary albumin levels were significantly decreased in the treatment group	Wei and Tan, (2005)
Diabetes and complications	53	41	94	T:23/30 C:41/18	T:38–62 C:36–65	DZXXI 20 ml,iv	Inosine 0.2 g, iv	14d	SOD and GSH-Px in the treatment group were significantly higher than those in the control group, while urinary microalbumin excretion rate (UAER) and ROS were significantly lower in the treatment group	Chen et al. (2007)
	30	30	60	T:16/14 C:17/13	T:46.4 ± 8.3 C:48.5 ± 8.6	DZXXI 30 ml,iv	Routine symptomatic treatment	14d	UAER, urinary fibrin degradation products (FDP), TC and TG were significantly decreased in the treatment group	Chen, (2009)
	45	45	90	T:25/20 C:29/16	T:56.9 ± 4.3 C:48.5 ± 8.6	DZXXI 30 ml,iv	Conventional treatment combined with compound 3D B (Ⅱ) 20 ml,iv	3w	Sensory nerve conduction velocity (SNCV) and motor nerve conduction velocity (MNCV) of each site in the treatment group were significantly better than those in the control group, and SOD and NO levels were significantly increased in the treatment group	Du et al. (2019)
	36	36	72	T:20/16 C:19/17	T:51–75 C:20–55	DZXXI 400 mg,iv	Routine insulin, antibiotics, vitamin B1,B12, sodium alginate 0.1 g, oral	14d	The total effective rate of the treatment group was 86.11%, and the whole blood viscosity, RBC aggregation index and RBC deformability index were decreased	Lin, (2008)
	48	20	68	T:26/22 C:12/8	T:61.5 ± 14.6 C:62.3 ± 11.5	DZHSI 20 ml,iv	Huangqi Injection 20 ml,iv	14d	The levels of TC,TG, PCV, PAGT, FIB and UAER were significantly decreased in the treatment group, while the level of HDL-C was increased	Kang and Liu, (2003)
	50	50	100	T:36/14 C:35/15	T:41–72 C:40–72	DZHSI 60 mg+normal saline 250 ml,iv	CT:Symptomatic treatment with conventional western medicine	15d	The treatment group significantly decreased the production of related inflammatory cytokines IL-6, TNF-α and hs-CRP in patients with early type 2 diabetic nephropathy	Li et al. (2011)
Liver disease	54	——	54	T:38/16	T:31–82	DZXXI 20 ml,iv	——	30d	In the treatment group, jaundice (TBIL) ALT, AST, R -- GT,HA, laminin, PⅢP, level of a C were significantly decreased	Wu et al. (2011)
	60	60	120	T:47/13 C:49/11	T:20–58 C:20–55	DZXXI 30 ml,iv	diammonium glycyrrhizinate 30 ml+Casilile300 mg,iv	30d	In the treatment group, the levels of HA, P-LP, TA-C, TGF-βandBIMP-1 were significantly decreased	Qiu, (2006)
	61	64	125	——	——	DZXXI iv	CT:comprehensive treatment	14d	In the treatment group, the level of albumin, prothrombin time activity and total bilirubin were restored	Liu, (2014)
	50	39	79	T:32/8 C:33/6	T:21–68 C:21–71	DZHSI 40mg, iv	Ganlixin injection + Yinzhihuang injection and Gantaille + vitamin C, vitamin B6, vitamin K1	4-8w	The total effective rate of 92.50% in treatment group was significantly higher than that of control group (74.36%), TBIL, ALT and AST levels were decreased	Hu et al. (2009)
Vertebrobasilar insufficiency	60	56	116	T:27/33 C:22/34	T:45–75 C:44–76	DZXXI 40 ml, iv	Betahistine Hydrochloride Injection 250 ml,iv	15d	The total effective rate of the treatment group was 91.67% higher than that of the control group (85.71%). The mean blood flow velocity of the vertebral artery and basilar artery was increased in the treatment group	Fang, (2008)
	70	70	140	T:34/36 C:33/37	T:45–75 C:43–76	DZXXI 40 ml,iv	Betahistine mesylate, 12 mg, take orally	14d	Vertebral artery peak flow velocity (VS) at the end of systolic period, peak flow velocity (VD) at the end of diastolic period and basilar artery VS were significantly increased in the treatment group	Xu et al. (2010)
	42	43	75	T:15/72 C:18/25	T:38–75 C:35–76	DZXXI 40 ml,iv	Buflomedil Hydrochloride Injection 0.15 g,iv	15d	The treatment group effectively reduced blood lipid and improved hemorheology, and the cure rate of symptoms was 90% higher than that of control group (82.5%)	Zhang et al. (2007)
	56	53	109	T:30/26 C:29/24	T:40–69 C:38–68	DZXXI 40 ml,iv	Routine symptomatic treatment	20d	The clinical efficacy of 94.64% was significantly higher than that of the control group (69.81%). The hemorheology indexes were improved, SOD level was increased and ox-LDL level was decreased	Zhu and Huang, (2009)
	35	35	70	T:21/14 C:19/16	T:17–71 C:15–69	DZHSI 20 mg,iv	Diphenhydramine Hydrochloride Tablets 25 mg,Diphenhydramine Hydrochloride Tablets 25 mg, Oral + buflomedil hydrochloride injection200 mg,iv	10d	The total effective rate of the treatment group was 94.29%, which was higher than that of the control group (71.43%)	Chen, (2011)
	140	140	280	T:64/76 C:68/72	T:54.3 ± 4.2 C:53.2 ± 3.3	DZHSI 100 mg,iv	Shenmai injection 30 ml,iv	14d	The mean flow velocity of the systolic and diastolic periods of the vertebral artery and basilar artery in the treatment group was faster than that in the control group, the pulse index (PI) was increased, and the vascular resistance was decreased	Yu, (2014)
Eye disease	20	20	40	——	T:18–69 C:17–68	DZHSI 20 ml,iv	Vitamin C Injection 10 ml,iv	14d	The visual field defect and retinal nerve fiber layer thickness were significantly decreased in the treatment group, and LP100 and AP100 were significantly improved in the treatment group	Du et al. (2016)
	83	64	147	——	——	DZXXI 50 ml,iv	Oral placebo	2m	The peak systolic velocity and end diastolic velocity resistance index of the central retinal artery of the posterior short ciliary artery were significantly thickened in the treatment group, and the brachial-retinal circulation time and retinal filling time were shortened.The area of optic disc along the optic disc increased significantly	Chang, (2018)
	42	42	84	——	——	DZXX Mistura 10 ml, oral	Citicoline Injection 2 ml,iv	20d	The total effective rate of the treatment group was 90.48% higher than that of the control group (73.81%). The visual field defect and P100 latency were significantly reduced, and the number of lines of visual acuity improved was increased	Dong, (2016)
Osteoarthritis of the Knee	20	19	39	T:9/11 C:8/11	T:45–65 C:42–66	DZXXI, 2000 ml, washing	Saline flush	21d	The clinical score of the treatment group was better than that of the control group, and the levels of IL-1 and TNF-α were significantly decreased	Yu et al. (2006)
	45	45	90	T:25/20 C:27/18	T:43–79 C:46–79	DZHSI,2 ml,washing	Sodium Hyaluronate Injection 2 ml,washing	35d	The total effective rate in the treatment group was 95.56%, which was significantly higher than that in the control group (77.78%). The Lysholm score was significantly increased, and the VAS score was significantly decreased	Liao, (2015)
Pancreatitis	35	35	70	T:19/16 C:24/11	——	DZHSI,20 mg	Local arterial perfusion of conventional drugs	——	The APACHEⅡ score, hs-CRP, IL6 and TNF-α in the treatment group were significantly lower than those in the control group, and the proportion of CD4 cells and the ratio of CD4/CD8 were higher than those in the control group	Zhang, (2015b)
	40	40	80	T:24/16 C:26/14	T:17–82 C:17–83	DZHSI 30 ml,iv+CT	Routine comprehensive treatment	14d	The levels of serum amylase, white blood cells, C-reactive protein, tumor necrosis factor-D and interleukin-1 were significantly decreased in the treatment group, and the levels of albumin, proalbumin, transferrin and hemoglobin were increased in the treatment group	Zhang et al. (2016)
	32	30	62	T:20/12 C:18/12	T:18–73 C:18–75	DZHSI 75–100 mg,iv	Continuous infusion of somatostatin with intravenous micropump, inhibition of gastric acid, supplementation of effective blood volume and nutritional support, and antibiotics	14d	In the treatment group,Cr,BUN, 24 h urinary protein levels, plasma TNF-α, IL-6, IL-8 levels and APACHEⅡ score were decreased	Bai et al. (2014)
Ear Disease	80	—	80	T:48/32	——	DZXXI 50 mg,iv	——	14d	The total effective rate was 91.25%	Su et al. (2009)
	20	—	20	T:12/8	T:22–65	DZXXI 40 ml,iv	——	20d	After treatment, the whole blood viscosity, plasma viscosity and hematocrit were significantly decreased	Liu and Cheng, (2007)
	30	30	60	T:16/14 C:16/14	——	DZHSI 50 mg,iv+ct	CT, Low molecular dextran,10%GS +ATP, Danshen tablets, vitamin B, prednisone tablets symptomatic treatment	14d	The clinical efficacy of the treatment group was higher than that of the control group, and the indexes of whole blood viscosity and platelet aggregation rate were significantly improved	Zhu et al. (2011)
Necrosis of the Femoral Head	23	21	44	T:18/5 C:18/3	T:28–58 C:20–58	DZXXI 40 ml,iv	Antibiotics to prevent infection	14d	The whole blood specific viscosity, plasma viscosity, hematocrit, erythrocyte aggregation index, erythrocyte deformability index, triglyceride and total cholesterol levels were significantly decreased in the treatment group	Pan et al. (2018)
	20	20	40	——	——	DZHSI 30–50 mg,iv	DSI 10∼16 ml,iv	20d	In the treatment group, the whole blood viscosity, plasma viscosity, RBC aggregation index decreased significantly, RBC deformability index and PO2 value increased significantly, and the pain was relieved	Zhang et al. (2004)
	28	—	28	T:10/18	T:22–55	DZXXI 20 ml, perfusion	——	——	Harris score increased and hip function improved after treatment	Ni et al. (2013)

T, Treatment group; C, Control group; DZXXI, Dengzhanxixin Injection; DZHSI, Breviscapine Injection; M/F, Male/Female; iv, intravenous injection; ig, intragastric administration; ip, intraperitoneal injection.

#### Cerebral Infarction

Wu divided 87 patients with cerebral infarction into a control group and an observation group. The control group was treated with compound Danshen injection, and the observation group was treated with DZXXI. After 14 days, DZXXI reduced plasma nitric oxide (NO) and plasma circulating endothelial cells (CEC) and endothelin (ET) levels in patients with cerebral infarction, improved the vascular endothelial function of patients with cerebral infarction, increased blood flow, and markedly improved microcirculation and nerve function (Wu et al., 2006). Another study also found that DZXXI can increase the expression of vascular endothelial growth factor (VEGF), endothelial progenitor cells (EPCs), brain-derived neurotrophic factor (BDNF), reduce serum adhesion molecules and platelet inflammatory factors, promote angiogenesis, and antiplatelet Gather to improve cerebral infarction (Zhen et al., 2012; Hou et al., 2015). In addition, breviscapine injection can also reduce the plasma tissue-type plasminogen activator (t-PA) in patients with cerebral infarction, improve blood lipids, and improve cerebral infarction (Zhang et al., 2008). Clinical data show that DZXX preparations are more effective than conventional antihypertensive, lipid-lowering, anticoagulant and other traditional Chinese medicine treatments. DZXX preparations can improve the patient’s blood rheology and endothelial cell function, reduce blood lipids and inflammation, and improve the prognosis of patients with cerebral infarction. The detailed study of DZXX and its related preparations in the treatment of patients with cerebral infarction is shown in Table 3.

#### Hyperlipidemia

68 patients with hyperlipidemia-related cerebral infarction were randomly divided into DZXXI group and compound Danshen injection group, and intravenous drip. After 14 days, the basic cure rate in the DZXXI group was 64.71%, which was significantly higher than the 32.35% in the compound Danshen group (*p* < 0.01). After treatment, the whole blood specific viscosity, plasma specific viscosity, fibrinogen, total cholesterol, and three Acylglycerol is significantly lower than before treatment (*p* < 0.05) (Wu et al., 2004). In clinical treatments, breviscapine injection has obvious curative effects on hyperlipidemia. After treatment, patients’ TC, TG, LDL-C, MDA, oxidized low-density lipoprotein Ox-LDL, and plasma endothelin (ET) significantly decrease. By contrast HDL-C, SOD, and NO increase to varying degrees. DZHSI improves hyperlipidemia by scavenging oxygen free radicals, lowering blood lipids and other activities, enhancing the activity of antioxidant enzymes in patients with hyperlipidemia, reducing lipid peroxidation damage, and protecting patients’ vascular endothelial function (Yu, 2011). The detailed study of DZXX preparations in the treatment of patients with hyperlipidemia is shown in Table 3.

#### Hyperviscosity

DZXX injection (observation group) and compound Danshen injection (control group) were used to treat patients with hyperviscosity. After the treatment, the results of DZXX injection reveal that whole blood viscosity, plasma viscosity, hematocrit, fibrinogen, cholesterol, triglyceride, and high-density lipoprotein cholesterol levels significantly improve (*p* < 0.05). The results of the study showed that the observation of HCT, Fib, TC, TG and HDL-C in observation group were significantly improved without adverse reactions compared with before treatment and after treatment (Chang, 2008). Therefore, DZXX injection can prevent and treat hyperviscosity by improving blood rheology, reducing fibrinogen, enhancing blood lipids, and prolonging the time of partial thromboplastin activity (Shen and Li, 2002). After 48 hospitalized patients with hyperviscosity were treated with DZHSI, the whole blood, high-shear viscosity, low-shear viscosity, plasma viscosity, fibrinogen, and other factors are significantly lower than the initial values (*p* < 0.01). This result indicates that the treatment can reduce blood viscosity, increase tissue perfusion flow, and effectively prevent a series of pathophysiological changes induced by hyperviscosity (Han et al., 2004). The detailed study of DZXX preparations in the treatment of hyperviscosity patients is shown in Table 3.

### Cardiovascular Diseases

Luo divided 60 patients with unstable angina pectoris into a treatment group and a control group. They were treated with DZXXI and compound Danshen injection. After 14 days, the clinical efficacy, electrocardiogram changes, and hemorheology of the patients in the DZXXI treatment group were all improved. The measured values of platelet CD41, CD63, CD62P and plasma D-dimer were significantly lower than the level before treatment, and the degree of improvement was more obvious than that of the control group, suggesting that DZXXI can improve angina pectoris by anti-platelet activation and improve coagulation and fibrinolysis activity (Luo, 2003). Other studies have also found that DZXXI can reduce the blood lipid level of patients with angina pectoris, improve lipid metabolism, reduce the expression levels of IL-6, CRP and TNF-α in the serum of patients, improve inflammation, and inhibit the development of angina pectoris (Zhao et al. (2008); Li and Yang, 2014). In addition, DZHSI can also inhibit platelet aggregation and internal coagulation function, activate the fibrinolytic system, promote fibrin degradation, interfere with related molecules before thrombosis, inhibit thrombosis, and thereby improve angina pectoris (Zhao et al., 2010). More clinical studies The data shows that compared with patients with angina pectoris who are given nitrate, anticoagulant, lipid-regulating western medicine and other traditional Chinese medicine compound injections, the symptoms of angina pectoris in patients treated with DZXX preparations are more improved, and the treatment efficiency is higher. The detailed study of DZXX preparations in the treatment of patients with angina pectoris is shown in Table 3.

### Pulmonary Diseases

DZXXI can significantly improve the clinical symptoms of patients with acute exacerbation of pulmonary heart disease, increase PaO_2_, decrease PaCO_2_, increase pH, significantly decrease plasma D-dimer and Hb (Li et al., 2006), and reduce plasma IL-8 and TNF-α, CRP, blood viscosity, hematocrit, and fibrinogen levels. The mechanism of action of DZXX injection may be related to reducing blood viscosity, cytoinflammatory factor levels, and blood hyperviscosity in patients with COPD, correcting heart failure, respiratory failure, and other factors, ensuring a stable lung function for elderly patients with moderate to severe COPD, and decreasing the number of acute exacerbations (Cao et al., 2006; Xiao et al., 2015). Other researchs also found that conventional treatment combined with adjuvant breviscapine injection reduced the level of serum endothelin (ET), soluble vascular cell adhesion molecule-1 (sVCAM-1), and soluble intercellular adhesion molecule-1 (sICAM-1) in patients with cor pulmonale, increase NO level and improve cardiac output (CO) and cardiac index (CI) (Kong et al., 2010). And In another study, it was found that DZHSI can enhance the body’s immune function and improve the therapeutic effect of patients with COPD (Li and Jia, 2015). These results indicate that breviscapine changes the vascular endothelial function of patients with chronic pulmonary heart disease, improves blood hypercoagulability and blood viscosity, and reduces the specific volume of blood cells. Consequently, this treatment reduces endothelial cell damage, restores the balance of vasoactive factors, minimizes platelet aggregation, lowers the occurrence of thrombosis, and improves heart function and clinical efficacy during decompensation (Kong et al., 2010). Detailed study of DZXX preparations in the treatment of pulmonary disease is shown in Table 3.

### Kidney Diseases

A number of clinical treatments have shown that both DZXXI and scutellarin injection mainly reduce serum creatinine (Scr), blood urea nitrogen (BUN), 24 h urine total protein (24 h UTP) and microglobulin (B2M) content, and Increase the creatinine clearance rate (Ccr) to play a therapeutic role and improve the patient’s renal function (Wu et al., 2018). In addition, it can also improve kidney lipid metabolism, blood rheology indexes and inflammatory factor levels to improve renal function (Cheng et al., 2005). The detailed study of breviscapine and its related preparations in the treatment of patients with nephropathy is shown in Table 3.

### Diabetes Complications

Patients with stage IV diabetic nephropathy were divided into treatment and control groups. The treatment group was injected with 20 ml of DZXX, and the control group was administered with 0.2 g of inosine once a day for 2 weeks and 1 course. The results show that DZXXI can significantly reduce proteinuria in patients with diabetes. DZXXI can reduce the urinary microalbumin excretion rate (UAER), increase SOD and GSH-Px activities, inhibit reactive oxygen species production and membrane lipid peroxidation, remove reactive oxygen species, increase tissue antioxidant enzyme activities, decrease ROS production through MAPK and JAK-STAT channels, reduce the thickening of the basement membrane of the diabetic glomerulus and the proliferation of the mesangial matrix, and delay glomerular sclerosis (Chen, 2009; Cheng et al., 2007). Kang used breviscapine injection to treat diabetic nephropathy and found that the urine albumin excretion rate of patients significantly decreases, and the hematocrit, platelet aggregation rate, and plasma fibrinogen significantly improve. These results suggest that breviscapine may reduce urine albumin in patients with diabetes, alleviate patients’ microcirculation disorders, and reduce blood viscosity (Kang and Liu, 2003). After 45 patients with diabetic peripheral neuropathy were treated with DZXXI, sensory nerve conduction velocity (SNCV) and motor nerve conduction velocity (MNCV) were restored, and the levels of SOD and NO were improved. By expanding blood vessels, removing oxygen free radicals, improving blood rheology indexes, and improving clinical efficacy (Du et al., 2019). The detailed study of DZXX preparation in the treatment of diabetic patients is shown in Table 3.

### Liver Diseases

DZXXI has a good curative effect on patients with chronic hepatitis B hepatic fibrosis. In particular, it reduces the serum indices of liver fibrosis especially the hepatic fibrosis indices hyaluronic acid (HA) and laminin (LN), improves the liver function and B-ultrasound imaging indices of the patients (Wu Y. H. et al., 2011), restores albumin level and prothrombin time activity (PTA), improves ALT, AST, and ALB and decreases the total bilirubin level of hyperbilirubinemia (Liu, 2014) Therefore, DZXX injection likely dilates small arteries, improves liver microcirculation and metabolism, and eliminates harmful substances, thereby improving liver function and reducing liver fibrosis (Wu D. F. et al., 2011). The detailed study of DZXX preparation in the treatment of liver disease is shown in Table 3.

### Vertebrobasilar Insufficiency

The 116 patients with vertebrobasilar artery insufficiency were randomly divided into a treatment group of 60 cases and a control group of 56 cases. The treatment group received intravenous infusion of DZXXI, and the control group received intravenous infusion of betahistine hydrochloride injection. Observed after 15 days of treatment, the total effective rate of the treatment group was 91.67%, and the total effective rate of the control group was 85.71%. The average blood flow velocity of the vertebrobasilar artery of the two groups was significantly improved, and the average blood flow velocity of the vertebral artery and the basilar artery were increased in the treatment group compared with the control group (*p* < 0.05) (Fang, 2008). Clinically, DZXX preparations can resist thrombosis, improve blood viscosity and microcirculation, and increase local blood supply. See Table 3 for detailed research.

### Eye Diseases

DZXXI can effectively improve the ocular hemodynamics of open-angle glaucoma patients with controlled intraocular pressure after selective laser trabeculoplasty (SLT), and improve the flow velocity and resistance index of the short posterior ciliary artery and central retinal artery), shorten the arm-retinal circulation time (A-CT) and retinal filling time (A-VT), increase the thickness of the retinal optic nerve fiber layer (RNFL), increase the area of the optic disc, improve the blood circulation of the optic disc, and improve the RNFL and optic disc edge Area, so as to protect the function of the optic nerve. The clinical studies are shown in Table 3.

### Osteoarthritis of the Knee

DZXXI is used clinically to treat knee osteoarthritis, which can effectively reduce cytokines in synovial fluid, remove pathogenic factors in joints, relieve pain and improve knee joint function, and improve the clinical symptoms and function of patients with knee osteoarthritis due to blood stasis block. The clinical studies are shown in Table 3.

### Pancreatitis

Clinically, breviscapine injection can effectively improve the nutritional status of patients with acute pancreatitis, increase the levels of albumin, prealbumin, transferrin, and hemoglobin, and reduce serum amylase, white blood cells, c-reactive protein, and tumor necrosis factor-α and interleukin-1 levels, improve immunity, and help patients recover (Zhang et al., 2016). Breviscapine injection can also reduce blood creatinine (Cr), urea nitrogen (BUN), 24 h urine protein levels, improve kidney function and inflammatory factors to improve pancreatitis symptoms (Bai et al., 2014). The clinical studies are shown in Table 3.

### Ear Diseases

Thirty patients with sudden deafness were treated with breviscapine injection. After 14 days, breviscapine injection was 70% effective, which was higher than 56.7% in the control group. Breviscapine injection significantly improves the blood viscosity of patients, resists platelet aggregation, improves clinical efficacy, and also greatly reduces the rate of deafness (Zhu et al., 2011). The clinical studies are shown in Table 3.

### Necrosis of the Femoral Head

Twenty-eight patients with femoral head necrosis (osteonecrosis of the femoral head, ONFH) were treated with DZXX injection perfusion in the internal circumflex femoral artery. Imaging showed that small branch vessels increase and elongate, the femoral head and neck vessels increase, and the area enlarges. The average Harris score before the treatment of the hip joint function was 64.9 ± 3.6, and the average after treatment was 76.3 ± 5.2. The difference was statistically significant (*p* < 0.05). The pain of the hip joint was reduced, the walking distance was increased, and the claudication was reduced. There were no obvious complications during follow-up (Ni et al., 2013). The clinical studies are shown in Table 3.

## Pharmacological Effects

**FIGURE 2:**
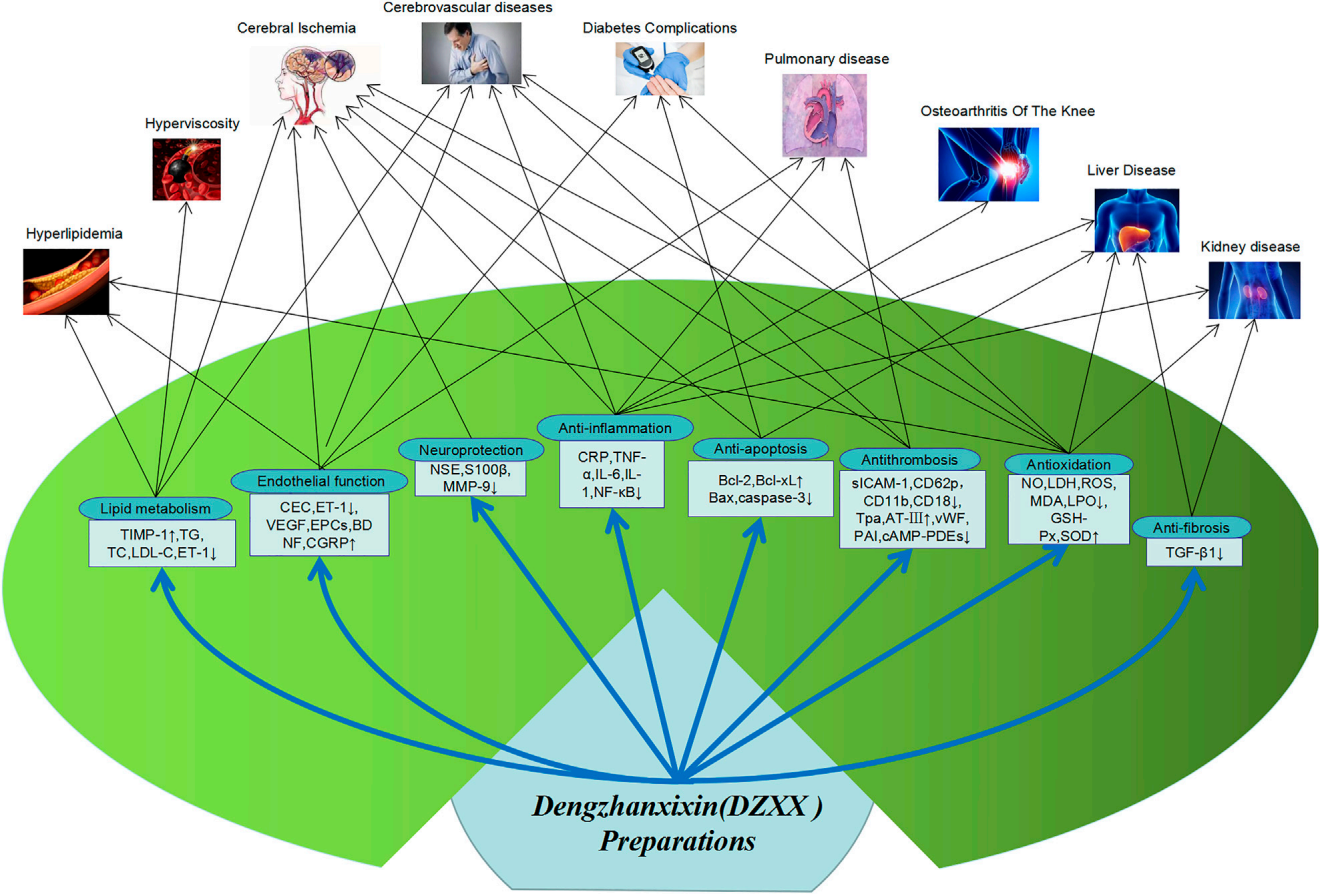
Main pharmacological action and action mechanism of DZXX preparations.

Looking at the literature, it is found that DZXXI, DZHSI, and DZHSP are the most commonly used DZXX preparations in pharmacological research. Therefore, the pharmacological researches on these three preparations and their main active ingredients are reviewed. And Main pharmacological action and action mechanism of DZXX preparation show in Figure 2.

### Dengzhanxixin Injection (DZXXI)

#### Cerebrovascular Diseases

Combined with clinical experimental data, it is found that DZXXI can reduce blood lipids, blood viscosity and serum adhesion molecules in the treatment of cerebrovascular diseases, thereby reducing platelet aggregation, increasing blood flow in brain tissue, improving blood supply, and reducing inflammatory factors TNF-α, IL-6 and the apoptotic factor caspase-3, thereby reducing the damage after cerebral ischemia and cerebral infarction (Wang JL. et al., 2005; Yin et al., 2017), and also improving hyperlipidemia and hyperviscosity. In the rat MCAO model, it was found that DZXXI up-regulated the expression of vascular endothelial growth factor (VEGF) and VEGF receptor Flk-1, induced angiogenesis in ischemic brain tissue, promoted damaged vascular endothelial repair, reduced EEG pathological spikes, increased the regional cerebral blood flow (rCBF) in the ischemic area (Guo et al., 2016). This may also be related to the significant reduction in NF-κB expression, inhibiting the formation or release of inflammatory mediators, and reducing nerve excitability, eliminating abnormal discharge after DZXXI treatment of cerebral ischemia and reperfusion in rats, thereby improving microcirculation, increasing oxygen supply to brain tissue, and reducing reperfusion injury after cerebral ischemia (Niu et al., 2007). In clinical and animal experiments, it has been found that DZXXI also upregulates brain-derived neurotrophic factor (BDNF), inhibits delayed neuron necrosis, and reduces cell apoptosis (Zhou Y. R. et al., 2011). In addition, in Liu’s research, it was found that DZXX may improve cell energy balance by regulating neuronal metabolism, maintain BBB stability after cerebral ischemia in rats, reduce brain edema, and play a role in brain protection (Liu et al., 2009). Related pharmacological studies are shown in Table 4.

**TABLE 4:** Pharmacological Effects of DZXXI preparations in various Diseases.

DZXX preparation	Disease	Animal/Cell	Model	Weight (g)	Dosage of administration/Culture concentration and method	Drug administration time/For days	Pharmacological action	Mechanism of action	References
DZXXI	Cerebrovascular disease	SD rat	MCAO	220–240	8.4 mg/kg, iv	after reperfusion	Anti-inflammatory, anti-platelet and neuroprotective	The serum level of TXB2 was significantly decreased	Zhao et al. (2019a)
		SD rat	MCAO	180–220	10 mg/kg, iv	10d	Repair vascular endothelial function	VEGF、VEGF mRNA、FLK-1、FLK-1 m RNA, rCBF significantly increase, EGG decrease significantly	Guo et al. (2016)
		Wistar rat	MCAO	250–300	45 mg/kg,ip	after reperfusion	anti-inflammatory	The expression of NF-κB p65 protein was decreased	Niu et al. (2007)
		SD rat	MCAO	270–320	2.7 mg/kg,ip	7d	neuroprotection	The expression of BDNF, trkB are up-regulated	Zhou et al. (2011b)
		Wistar rat	MCAO	200–250	22.5 mg/kg,ip	7d	protect BBB	NAA/Cr ratio to reduce,Cho/Cr ratio increases	Liu et al. (2009)
	Cardiovascular disease	SD rat	Hypertension	193.3–343.5	10 mg/kg,ip	8w	decompression, refrain PKC	LVW/BW significantly decrease,Ⅰ type Collagen to reduce, Inhibits vascular smooth muscle cells PKC	Zhou et al. (2002b)
		SD rat	AMI	200–250	10,30,50 mg/kg,ip	7d	antithrombus	restrain TNF-α and PAI-1expression, stimulate the elevation of tPA expression, significantly decrease LVEDP, significantly increase MAP、LVPmax and±dp/dt	Huang et al. (2010)
		SD rat	Hypoxia	200–300	4 mg/kg,ip	4w	antioxidant	SOD activity increased significantly, MDA content decreased significantly	Li and Zhang, (2010)
		CMEC	AMI	——	12.5, 25, 50, 100 mg/L,ip	——	anti-inflammatory	LDH,IL-6 m RNA,MCP-1,MCP-1mRNA express to reduce	Zhang et al. (2009a)
	Pulmonary disease	SD rat	Hypoxia	200–300	4 ml/kg, ip	4w	antioxidant	The activity of SOD decreased, MDA content is higher	Li and Zhang, (2010)
		SD rat	Hypoxia	200–250	50 mg/kg,ig	3w	Decompression, Antivascular remodeling	mPAP、Hgb、HCT reduce, Collagen type I expression, endothelial ET-1 decreased	Su et al. (2006)
		SD rat	Hypoxia	280–320	25, 50 mg/kg,ip	3d	anti-inflammatory	Level of MPO and NF-κB decreased significantly	Chen et al. (2008)
	kidney disease	SD rat	5/6 nephrectomize	180–200	6 ml,3 ml,1.5 ml/kg, ip	12w	Antioxidant and anti-inflammatory	The contents of TNF-α and BUN were decreased, increasing SOD and CRE content	Ren et al. (2009)
		SD rat	IRI	——	12 ml/kg,ip	14d	Antioxidant and anti-apoptotic	BUN, SCr, MDA, BAX decrease, SOD, BCL-2 increase	Wang et al. (2014)
		SD rat	IRI	250–300	1.2 ml/100 g,iv	15 min before ischemia	Antioxidant	SOD activity increased, MDA content decreased	Yang et al. (2012)
DZHSI	Cerebrovascular disease	SD rat	MCAO	220–240	50 mg/kg,iv	onset of reperfusion	Up-regulating the expression of Nrf2/HO-1 pathway, antioxidant	Decreased levels of 4-HNE and 8-OHdG, raised the expression of Nrf2 and HO-1 proteins	Guo et al. (2014)
		SD rat	MCAO	180–190	1 mg, iv	14d	Inhibits the thrombin activation pathway	NO,eNOs, tPAT activity and PGI2 expression increase, clotting time, thrombin time prolong, TXA2 content, PAI activity, Internal/exogenous coagulation factors Xa and thrombin content decreased	Zhu et al. (2018)
		Wistar rat	MCAO	150–200	1.5 mg/kg,ip	3d	Neuroprotection	NAA, Cre elevate, the density of Cho and Glu goes down	Chen et al. (2007)
		Gerbils	MCAO	50–60	90 mg/kg,ip	15 min before ischemia	Inhibitor energy metabolism disorder, relieve brain edema	Restrain ATP and the adenosine pool content decreased	Wang et al. (2005b)
		SD rat	MCAO	250–280	0.33 mg/kg, i.p	7d	Neuroprotection	Decreased LC3 expression levels in neurons and astrocytes	Zhang et al. (2017a)
		SD rat	MCAO	280–300	75 mg/kg, ip	14d	Anti-apoptosis	the expression of caspase-3decreased	Hao et al. (2017)
		SD rat	CCI	260–280	2.5,5,10 mg/kg,ip	4w	Anti-inflammatory and anti-apoptotic	Inhibitory activation of NLRP3 inflammasome in hippocampus,down-regulation of caspase-1, IL-6 and IL-1β protein expression, inhibits the activation of caspase-3 protein and inhibits neuronal apoptosis	Wang et al. (2020)
	Cardiovascular disease	SD rat	MIRI	200–300	2,4,8 mg/kg,ip	20 min before ischemia	Eliminate oxygen free radicals and inhibit lipid peroxidation	Decrease LDH in blood and MDA in myocardium, protects myocardial SOD activity	Liu et al. (2008)
		SD rat	MIRI	200–240	25, 50 mg/kg, ip	7d	Anti-inflammation	The levels of TNF-α and IL-6 in serum of rats were significantly decreased after MIR	Gong et al. (2013a)
		SD rat	MIRI	200–240	25, 50 mg/kg, ip	7d	Antimitochondrial apoptosis	The expression level of Caspase-9 protein and mRNA descreased	Gong et al. (2013b)
		SD rat	MIRI	200–240	25,50 mg/kg, ip	7d	Regulat NF-kB pathway, anti-apoptosis	IKB-α protein was significantly reduced, P65 and α7nAChR proteins were significantly increased	Ding et al. (2018)
		SD rat	MIRI	200–240	25,50 mg/kg, ip	7d	anti-apoptosis	reduce the Caspase-3 gene expression	Gong et al. (2011)
		SD rat	MIRI	200–240	25,50 mg/kg, ip	7d	anti-apoptosis	STAT1 protein expression was significantly reduced, STAT3 protein expression was significantly enhanced	Han et al. (2012)
		C57	MIRI	22–28	50 mg/kg, ip	7d	anti-apoptosis	The expression of miR-140 was up-regulatedInhibitory expression of caspase3 mRNA, TLR4 and NF-κB protein	Wang et al. (2017)
		Rabbit	MIRI	2000–2,500	5 mg/kg	5 min before ischemia	Improves energy metabolism	LA and NEFA levels in serum and myocardial tissue were decreased, ATP was increased	Lu and Jiang, (2010)
	Pulmonary disease	SD rat	pulmonary fibrosis	200 ± 10	10 mg/kg,iv	20d	antioxidant	reduce the levels of MDA and HYP, increase the level of SOD	Li, (2009)
		BALB/c mice	NSCLC	20–22	10,20 mg/kg, ip	21d	Promote apoptosis of cancer cells	The expression of Bax was up-regulated, the expression of Bcl-2 was inhibited, Caspase-3 was activated	Wei et al. (2020)
		Wistar rat	COPD	——	10 mg/kg, ig	28d	Anti-fibrosis	MMP-9, TGF-and Smad 3 mRNA decreased and Smad 7 mRNA increased	Du et al. (2017)
	Kidney disease	Wistar rat	UUO	200–300	100 mg/kg, ip	14d	anti-fibrosis	The protein expression levels of TGF-β1 and α-SMA were decreased	Yang et al. (2009b)
		SD rat	AKI	200–250	20 mg/kg, iv	3d	-	The expressions of Na+,K+ -ATPase were up-regulated	Chen et al. (2014)
		Kunming mice	KI	18–22	25 mg,50 mg/kg,ig	7d	Protein to repair	The activity ofUPR/UCR and NAG-U, expression of TGF-β1 was decreased, and the expression of Ⅳ type collagen (Col-A) was enhanced	Li et al. (2013)
		Kunming mice	KI	18–22	25 mg,50 mg/kg,ig	7d	antioxidant	The levels of BUN and SCR were significantly decreased, and the levels of MDA in renal cortex were decreased、SOD and glutathione peroxidase levels increase	Lou et al. (2015)
		SD rat	ON	200–240	100, 200 mg/kg, ig	7d	Activation of blood vessels	The contents of ET-1, ACE and AngⅡ decreased, while NO increased	Ren et al. (2013)
	Diabetes and complications	SD rat	DN	180–220	10,15,20 mg/kg, ip	8w	reduce nephridial tissue VEGF expression	The renal index, UAER, Scr and BUN were significantly decreased, and the expression of VEGF in renal tissue was decreased	Cui and Liu., (2016)
		Wistar rat	DM	200–250	20 mg/kg, ip	4w	Activation of blood vessels	Reduces nitric oxide and angiotensin Ⅱ level	Gao et al. (2007)
		SD rat	DN	180–200	60 mg/kg,ip	12w	degrade extracellular matrix	The expression of MMP-9 was increased and BUN and Scr were decreased significantly	Deng et al. (2014)
		Wistar rat	DPN	200–300	20 mg/kg, ig	4w	neural restoration	SNCV and MNCV to increase	Xie and Li, (2010)
		Wistar rat	DM	160–200	10 mg/kg, ip	3d	Improve blood flow and fight fibrosis	UAER and NO to reduce, ADM, ADMR mRNA and protein expression to increase	Chen et al. (2011)
		SD rat	DM	200–250	20 mg/kg, ip	6w	anti-apoptosis	the expression of bcl-2mRNA was enhanced, baxmRNA was weakened,bax/bcl-2 ratio was reduced	Zhao et al. (2009)
		SD rat	DM	——	20 mg/kg, ip	16w	anti-angiogenesis	The protein expression levels of VEGF, VEGFR2 and p-ERK in ARPE-19 cells were decreased, and the expression of VEGF in retina of diabetic rats was inhibited	Wang et al. (2015a)
DZHSP	Cardiovascular disease	SD rat	AS	180–220	2 ml/kg,ig	12w	Improve hemorheology and hemodynamics	Hemorheological indexes, TC, TG, LDL-C contents were significantly decreased, and HDL-C was significantly increased	Zhao et al. (2017)
		SD rat	AS	180–220	6,12,24 mg/kg, ig	12w	Lower blood lipid, anti-inflammatory, anti-oxidation	TC、TG、LDL-C,CRP、TNF-α、IL-1β、IL-6, MDA content significantly reduced,SOD、CAT significant increase in activity	Wu et al. (2017)
		SD rat	SID	220–240	50 mg/kg, ig	30d	Anti-apoptotic, regulate JAK pathway	Bax、Caspase3、Caspase6 and JAK1、JAK2、JAK3、TIMP1、TIMP2、TIMP4、GRP78 and CHOP protein expression level decreased,TGFβ、Bcl2 protein expression increased	Shin et al. (2018)
	Liver disease	Wistar rat	LF	180–220	80 mg/kg, ig	8w	anti-fibrosis	Decreased the expression of TGF-β1 and Smad3	Zhong et al. (2016)
		Wistar rat	LI	172–225	20 mg/kg, ig	7d	antioxidant	SOD activity increased, MDA content decreased, ALT and AST content decreased	An et al. (2013)
		Mice	LI	21–25	40,80 mg/kg, ig	14d	antioxidant	decreased liver index, ALT activity, MDA content, and significantly increased SOD activity	Yang et al. (2011)
		Wistar rat	LF	200–250	4,8,12 mg/kg, ig	4w	——	TIMP-1 was decreased and MMP-13 was increased	Gan et al. (2012)
	Pulmonary disease	SD rat	Hypoxia	200–280	50 mg/kg, ig	4w	Regulates the PKC signaling pathway	PKCt、PKCm、PKCc and the percentage of PKCM in PKCT decreased significantly	Zhou et al. (2002a)
		SD rat	Hypoxia	200–280	50 mg/kg, ig	4w	depressurization	To reduce the mRNA expression of Ⅰ type procollagen in the wall of the tube	Chen et al. (2002)
	Kidney disease	Wistar rat	AKI	230–250	6,12,24 mg/kg, ig	14d	Antioxidant and anti-inflammatory	serum BUN、Scr,KIM-1 expression,MDA,MIP-2,MIP-1,TNF-α content decreased,SOD, CAT level enhanced	Zhao et al. (2019b)
		SD rat	IRI	250–300	6 ml/kg, ip	Preoperative 30 min	Antioxidant and anti-apoptotic	The expression of L-selectin protein was decreased, SOD activity was increased, NOS, NO and MDA levels were decreased	Jia et al. (2011)
	Diabetes and complicationss	Wistar rat	DN	180–220	100 mg/kg, ig	120d	antioxidant	SOD activity increased and LPO content decreased	Han, (2009)
		C57/BL6J	Nephropathy	——	10 mg/kg, ig	4w	suppression of renal fbrosis	decreased phosphorylation of PKCβII, Akt, JNK1/2 and p38	Jiang et al. (2016)
Scutellarin	Cerebrovascular disease	SD rat	MCAO	140–180	25 mg/kg, iv	onset of reperfusion	Anti-apoptosis	Brain tissue damage was reduced, the expression of Bcl-2 increased and the expression of Bax decreased	Yang et al. (2019a)
		Wistar rat	MCAO	270–300	12.5, 25, 50 mg/kg, iv	after reperfusion	Anti-apoptosis	The expression of HSP70 protein and mRNA was increased	Wang, (2015b)
		SD rat	MCAO	140–180	4 ml/kg, iv	12 h after reperfusion	Protect BBB	The expression level of MMP-9 and MMP-9 mRNA and the permeability of BBB were decreased	Yang et al. (2019b)
		Wistar rat	MCAO	270–300	12.5, 25, 50 mg/kg, iv	after reperfusion	Anti-apoptosis	Restrained Cas-pase-3mRNA and protein expression	Wang et al. (2006b)
		SD rat	MCAO	250–280	20,40、60 mg/kg,ip	30 min before ischemia	Neuroprotection	Iimprove NGF, BDNF and GDNF mRNA expression	Chai et al. (2013)
		SD rat	MCAO	220–250	50,75 mg/kg, ig	7d	Anti-apoptosis	Reversed brain NAD depletion, reduced DNA fragmentati, inhibited PARP overactivation and AIF translocation	Zhang et al. (2009b)
		SD rat	MCAO	250–280	100 mg/kg, ip	60 h	Anti-Infammatory	Attenuated the expression of p-p38, p-JNK, iNOS, TNF-α, and IL-1β, increased p-ERK1/2	Chen et al. (2020)
		SD rat	pBCAO	280–300	15,30 mg/kg, iv	28d	Anti-inflammatory	Suppress Aβ formation, microglial activation and APP,BACE-1 expression	Jung et al. (2018)
		SD rat	MCAO	230–280	25,50,75 mg/kg, ig	7d	Neuroprotection	Upregulate eNOS expression and downregulate VEGF, bFGF, and iNOS expression	Hu et al. (2005)
	Alzheimer’s diseaseAlzheimer’s disease	Kunming mice	Dementia	——	40 mg/kg,ig	90d	Histone modification	The expression of histones (H2a 1-H, H2a.2, H2a.Z, H3.3c) was decreased	Guo et al. (2017)
		Wistar rat	Dementia	300–350	2 ml,ig	20d	Reduces oxidative stress and antiapoptosis	It increased the activity of SOD and decreased the activity of MAO and the incidence of nerve cell apoptosis	Guo et al. (2011a)
		Wistar rat	Dementia	300–350	2 ml,ig	20d	Neuroprotection	The expression levels of α4 and α7 nAChR were up-regulated	Guo et al. (2011b)
		Wistar rat	Dementia	300–350	2 ml,ig	20d	Protects the cholinergic system	The activities of AChE and BuChE in brain tissue homogenate decreased, while the activities of AChE in plasma increased	Xie et al. (2011)
		Wistar rat	Dementia	300–350	2 ml,ig	20d	Anti-inflammatory	The expression of NF-κB p65 mRNA was down-regulated, and the expression of IL-1β, IL-6 and TNF-α in positive cells was decreased	Guo and Guan, (2013)
		C57BL mice	AD	——	15,25,40 mg/kg, ig	90d	Regulate APP metabolism	decreased APP、BACE1 protein and mRNA expression and enhanced BACE2 protein expression	Wang et al. (2018)
		SH-SY5Y cell	AD	——	——	2d	Anti-apoptosis	Decreased the protein and mRNA expressions of 1, 4, 5-triphosphate inositol receptor (IP3R), Bax and caspase-3, and increased the protein and mRNA expressions of Bcl-2	Wang et al. (2019)
		SH-SY5Y cell	AD	——	——	2d	Anti-neurotoxic injury	The expression levels of Aβ1-42, APP, BACE1 protein and M RNA were decreased, and the expression levels of BACE2 protein and M RNA were increased	Ao et al. (2018)
	Cardiovascular disease	HCMEC cell	IR	——	1μ,10 μmol/L	——	Antioxidant and anti-apoptosis	The content of MDA was decreased and the expression of P-ERK1/2 protein was increased	Huang et al. (2017)
		SD rat	MIRI	280–350	0.3, 3,30 mg/kg, ip	7d	Anti-inflammatory	The concentrations of ICAM-1, IL-1β, IL-6, IL-18 and TNF-α were decreased, the activities of AST, CK and LDH were increased, and the NF-R/NLRP3/IL-1β pathway was inhibited	Ji et al. (2019)
		Minipig	AS	10,000–15,000	1.5, 3, 6 mg/kg,ip	12w	Stable plaque	The mRNA and protein expressions of MMP-1, 2 and 9 were down-regulated	Zhang et al. (2020)
		H9C2 cell	IR	——	25, 50, 100 μmol/L	——	Antiapoptosis, anti-inflammation, anti-oxidation	restrain TNFα,IL-1β,IL-6,IL-8,CK,ROS,MDA, increased SOD	Wang et al. (2016)
		SD rat	HFD	180–220	5, 10,20 mg/kg, ig	6w	Reduces blood lipid and is anti-oxidant	enhanced SOD and NO, lowered TC, TG, LDL-L, MDA level	Mo et al. (2018)
		SD rat	MIRI	250–280	5, 10,20 mg/kg, ip	15 min before ischemia	Anti-inflammatory	reduced NLRP3 inflammasome activation, inhibited mTORC1 activity, and increased Akt phosphorylation	Xu et al. (2020)
		SD rat	AS	180–220	6.25,25 mg/kg, ip	——	Reducing blood lipid, anti-oxidation and anti-apoptosis	increased VCAM-1, ICAM-1, IL-6, TNF-α IL-10, MDA, ROS levels, enhanced SOD, CAT, T-AOC	Fu et al. (2019)
		SD rat	AS	180–220	5, 10,20 mg/kg, ig	6w	Antioxidant, improve dyslipidemia, maintain the balance of fibrinolytic system	increased SOD,NO,HDL-C, tPA levels, reduced MDA,TG、TC and LDL-C,PAI-1 levels	Zhang et al. (2017b)
	Liver disease	ICR mice	LI	18–24	50,100 mg/kg, ip	1,9 h before modling	Anti-inflammatory	The levels of ALT, AST, NO2/NO3 and TNF-a were decreased, and the mRNA expressions of TNF-a and iNOS and the protein expressions of c-fos, c-jun and iNOS were down-regulated	Tan et al. (2007)
		ICR mice	LI	18–22	7.5,15 mg/kg,ig	5d	Antioxidant stress	Reduced ALT/AST ratio,ALP, MPO activity, TNF-α, IL-6,IFN-γ, MDA levels, enhanced GSH level	Niu et al. (2015)
		HepG2 cells	LC	——	3, 10, 30, 100 μM	3d	apoptosis-promoting	Reduce ROS and STAT3 protein	Xu and Zhang, (2013)
		C57BL/6 mice	HF	18–22	12.5, 25, 50 mg/kg, ig	10w	hypolipidaemic, antioxidative, and liver protective	Activate PPARc, PGC-1a, Nrf2, HO-1, GST, NQO1, restrain NF-kB, Keap1	Zhang et al. (2018)
		Kunming mice	LI	18–22	50, 100, 200 mg/kg, ig	12d	Antioxidant, scavenging free radicals	Decreased the activity of ALT, increased the activities of CAT, SOD, GSH-Px and decreased the contents of XOD, MDA, NO in liver tissue homogenate	Yang and Guo, (2010)
		SD rat	LF	150–180	25,50,100 mg/kg, ig	4w	Anti-fibrosis, improve liver function	Decreased the contents of ALT, AST, TBIL, HA, LN, CⅣ in rat serum, increased the contents of ALB, TP, and decreased the contents of C in rat liver tissue	Wang et al. (2015)
	kidney disease	C57BLKS/J mice	db/db	——	25,50,100 mg/kg, ig	8w	Reduces blood lipid and is anti-oxidant	up-regulated Nrf2, HO-1, SOD,GSH,CAT,HDL-C, down-regulated GSK,ICAM2,TC	Liu et al. (2019)
		ACHN and 786-O cell	HCC	——	30,60, 90 μM	3d	Propelling apoptosis and anti-tumor	The expression of cyclin D1, CDK2, Bcl2, MMP-2 and MMP-9 were decreased, and the expression of Bax, cleaved caspase 3 and p21 were increased	Deng et al. (2018)
		C57BL/6 mice	RI	20–23	30,60 mg/kg,ig	5d	Anti-inflammatory and anti-apoptotic	Decreased TNF-α and IL-6 levels, Cleaved caspase-3, Cleaved PARP, p53 expression, and Bax/Bcl-2 ratio	Sun et al. (2019)
		C57BL/6 mice	HN	25–27	5, 10,20 mg/kg, ip	3w	Anti-inflammatory and anti-apoptotic	decreased Scr, BUN, NGAL, Kim- 1, cystatin C, and IL-18 protein expression levels	Li et al. (2020)
	Diabetes and complications	Wistar rat	T2DM	180–220	50, 100, 150 mg/kg, ig	8w	Antioxidant and anti-inflammatory	the expression level of TNFα,IL-6,IL-1β,MDA,Caspase-9 and Bax was reduced, the GSH, SOD content and Bcl-2 expression were increased	Liu et al. (2018b)
		SD rat	T2DM	230–250	40 mg/kg,ig	8w	antiangiogenic effects	suppressed the crosstalk of phospho-ERK, phospho-FAK, phospho-Src, and VEGF	Long et al. (2019)
		Wistar rat	DM	200–250	100 mg/kg, ig	10w	prevented endothelial dysfunction	inhibits the translocation of PKC	Su et al. (2012)
		C57BL/6 mice	DR	18–22	5, 10 mg/kg, ig	1m	Antioxidant and anti-inflammatory	restrain NF-κB activation,TNF-α, ROS expression, ERK1/2, enhance claudin-1 and claudin-19,Nrf2	Mei et al. (2019)
		C57BL/6 mice	DM	18–22	50 mg/kg,ig	16w	Lower blood sugar and blood lipid, and resist oxidation	increased glycogen content,SOD, GSH-Px, CAT, GSH and T-AOC, reduced MDA, G6Pase and PEPCK enzyme activity	Gao et al. (2020)

#### Cardiovascular Diseases

Combined clinical trials have found that DZXXI can also improve heart blood supply and angina pectoris by reducing adhesion molecules and platelet aggregation, improving blood viscosity and blood lipids. In addition, in animal and cell experiments, it has been found that DZXXI can reduce the release of inflammatory factors IL-6 and MCP-1 in cardiac microvascular endothelial cells (CMEC) induced by TNF-α stimulation, and protect vascular endothelial cells by inhibiting inflammation (Zhang et al., 2009). It was also found in the rat model of myocardial infarction that DZXXI can directly inhibit the overexpression of TNF-α, regulate the balance of PAI-1 and tPA, slow down thrombosis, and improve the hemodynamics of the heart after infarction (Huang et al., 2010). DZXXI can also reduce the content of MDA in the heart and lung tissues of hypoxic rats, increase the level of SOD, and improve heart function by scavenging oxygen free radicals (Li and Zhang, 2010). Related pharmacological studies are shown in Table 4.

#### Pulmonary Diseases

In the COPD rat model test, it was found that DZXXI can reduce rat hematocrit, reduce the expression of pulmonary arteriole type collagen and endothelin 1 (ET-1), reduce pulmonary vascular resistance, thereby inhibiting pulmonary vasoconstriction and remodeling, relieving pulmonary hypertension and preventing the formation of hypoxic pulmonary hypertension (Zhang et al., 2009), which is also consistent with the previous literature reports of clinical therapeutic effects. DZXX can improve the proportion of blood flow in the lungs and increase the partial pressure of arterial blood oxygen, improve hypoxemia, reduce hematocrit, and reduce pulmonary vascular resistance (Li et al., 2006; Xiao et al., 2015). In addition, DZXXI can also reduce the content of MDA, increase the expression of SOD, improve lung hypoxia and improve lung function through anti-oxidation (Li and Zhang, 2010). Animal experiments have also found that DZXXI can reduce neutrophil infiltration in lung tissue and inhibit inflammatory damage by inhibiting NF-κB activation. This is also in line with the reduction of TNF-α, IL-8, CPR and other inflammatory factors in patients with COPD in clinical trials (Chen et al., 2008). Related pharmacological studies are shown in Table 4.

#### Kidney Diseases

Consistent with clinical treatment data, DZXXI also effectively reduces the animal’s BUN and Scr levels in the treatment of animal kidney disease models and improves renal function. A number of experimental data show that DZXXI can reduce the level of TNF-α, increase the activity of Bcl-2 and SOD, reduce the content of BAX and MDA in the animal kidney injury and renal insufficiency model. DZXXI protects the kidney by reducing kidney inflammatory factors, scavenging oxygen free radicals, and reducing renal tissue cell apoptosis. Related pharmacological studies are shown in Table 4.

#### Others

##### Eye Diseases

DZXXI can promote the recovery of optic nerve axoplasmic transport block in acute experimental high intraocular pressure rats. After 20 days of administration, it was found that axoplasmic transport was partially restored, and the number of retinal ganglion cells (RGCs) increased, thereby protecting the optic nerve (Li Q. C. et al., 2007). In addition, DZXXI was intraperitoneally administered to glaucoma rats. After 7 days, it was found that the density in RGCs and the thickness of layers in retina increased, which had a certain protective effect on experimental glaucoma in rats (Zhu et al., 2000; Li Q. C. et al., 2007). Related pharmacological studies are shown in the Table 4.

##### Diabetes Complications

Studies have also found that injecting DZXX into the vitreous of diabetic mice can increase the GAP-43 protein and promote the regeneration of the optic nerve in diabetic mice (Tian et al., 2020). In addition, combined with previous clinical studies, DZXXI increases the activity of SOD and GSH-Px in the renal tissues of patients with diabetic nephropathy and reduces the production of related inflammatory factors IL-6, TNF-α and hs-CRP, inhibit the production of reactive oxygen species and membrane lipid peroxidation and reduce renal inflammation, thereby delaying glomerular sclerosis (Cheng et al., 2007; Li et al., 2011). In addition, studies have found that DZXXI can also up-regulate the protein expression of MMP-9 in kidney tissue to achieve the purpose of treating diabetic nephropathy (Wang and Li, 2006). Related pharmacological studies are shown in the Table 4.

### Breviscapine Injection (DZHSI)

#### Cerebrovascular Diseases

In an MCAO (Middle cerebral artery occlusion) rat model, DZHSI can inhibit neuron-specific enolase levels, 4-hydroxy-2-nonenal and 8-hydroxy-2-deoxyguanosine and increase the expression levels of nuclear factor red sample 2 related factor (Nrf2) and heme oxygenase-1 (HO-1) protein. These results suggest that DZHSI may increase the expression of the Nrf2/HO-1 pathway and play a role in the treatment of cerebral ischemia-reperfusion injury (Guo et al., 2014). DZHSI can also prevent the activation of protein kinase C (PKC) induced by cerebral ischemia and reperfusion, reduce calcium overload, and decrease the volume of ischemic infarction (Chen and Dong, 1998; Shuai and Dong, 1998). DZHSI is used in advance on forebrain ischemia–reperfusion model gerbils, and results reveal that pre-administration before ischemia can significantly inhibit the decrease in hippocampal ATP and adenylate pool content caused by cerebral ischemia–reperfusion and reduce the water content of the brain cortex, the mechanism of action may be related to the reduction of energy metabolism disorders and cerebral edema caused by cerebral ischemia and reperfusion (Wang J. G. et al., 2005). DZHSI can also inhibit the production and release of interleukins and other inflammatory factors and caspase and other apoptotic factors, inhibit the activation of NLRP3 inflammasomes in hippocampus, and reduce the pathological damage and apoptosis of ischemic neurons (Hao et al., 2017; Wang et al., 2020). Related pharmacological studies are shown in the Table 4.

#### Cardiovascular Diseases

The clinical treatment results of DZHSI also show that it can improve blood viscosity and blood lipids in patients with angina pectoris, reduce the expression of prethrombotic molecular markers, reduce the platelet aggregation rate, and protect vascular endothelial cells. Animal experiments have found that high-fat feeding combined with the intravenous injection of calf serum albumin can be used to establish an atherosclerosis model in rabbit. Treatment with DZXXI can increase plasma vasodilator, HDL-C, TIMP-1 levels, reduce TC, TG, LDL-C, vasoconstrictor (ET-1), and MMP-9 levels, decrease plaque lesions, and delay plaque progression. These results indicate that DZXX injection can regulate lipid metabolism, may improve vascular endothelial functions, and adjust the balance of MMP-9/TIMP-1, thereby reducing atherosclerotic plaque (Cai et al., 2017). It was found that in animal models of myocardial ischemia, DZHSI mainly reduces myocardial cell apoptosis and relieves myocardial ischemia by down-regulating apoptotic factors such as caspase 3 and STAT1. In addition, DZHSI can also reduce the myocardial injury of ischemia-reperfusion rats by inhibiting inflammatory factors such as TNF-α (Han et al., 2012; Wang et al., 2017). Related pharmacological studies are shown in the Table 4.

#### Pulmonary Diseases

DZHSI can inhibit the increase in bronchial wall thickness and collagen fiber thickness in COPD model rats, reduce MMP-9, TGF-and Smad3 mRNA levels in lung tissue in COPD rats, raise Smad7 mRNA levels and improve fibrosis in lung tissue, thus delaying or improving the disease progression of COPD airway remodeling (Du et al., 2017). In addition, DZHSI can also improve lung tissue fibrosis by increasing lung tissue SOD content, reducing MDA level, and removing oxygen free radicals in lung tissue (Li, 2009). At the same time, DZHSI can reduce the content of Bcl-2 in lung cancer cells and increase the expression of Bax and Caspase 3 to promote the apoptosis of non-small cell lung cancer A549 CELL and play an anti-cancer effect (Wei et al., 2020). Related pharmacological studies are shown in the Table 4.

#### Kidney Diseases

In the rat model of kidney injury, after DZHSI administration, the levels of BUN, Scr, and MDA are reduced, SOD activity is increased, and kidney injury is improved through anti-oxidation (Lou et al., 2015). This is consistent with the results of clinical trials. Experimental studies have found that DZHSI can affect vasoactive substances in renal tissues, down-regulate the expression of transforming growth factor (TGF)-β1 and α-SMA proteins, increase the level of type IV collagen, and reduce renal damage due to renal fibrosis (Li et al., 2013). Related pharmacological studies are shown in the Table 4.

#### Diabetes and Complications

DZHSI reduces the expression of VEGF, the levels of nitric oxide and angiotensin Ⅱ in the kidney tissue of diabetic nephropathy rat models, protects vascular endothelial cells, activates blood vessels and improves renal hemodynamics (Gao Y. et al., 2007; Cui and Liu, 2016). In addition, studies have also found that DZHSI improves renal cell apoptosis through its anti-apoptotic effect. Other studies have shown that DZHSI can inhibitthe expression of MMP-9 and reduce the deposition of the mesangial matrix, thereby blocking the occurrence and development of diabetic nephropathy (Deng et al., 2014). Zhao found that DZHSI inhibited renal cell apoptosis by affecting the expression of apoptosis-related genes bcl-2 and bax, thus exerting a renal protective effect (Zhao et al., 2009). Similarly, for retinopathy caused by diabetes, DZHSI can also delay the course of diabetic retinopathy by reducing the expression of VEGF in the rat retina (Wang Y. H. et al., 2015). Related pharmacological studies are shown in the Table 4.

#### Liver Diseases

*In vivo* and *in vitro* experiments have demonstrated that breviscapine significantly reduces ALT, AST, and hydroxyproline levels in a dose-dependent manner. Breviscapine inactivates CCl_4_ and LPS-induced MAPK (p38, ERK1/2 and JNK) signals and Toll-like receptor 4 (TLR4)/nuclear factor-κB (NF-κB) signaling pathway, downregulates the expression and chemokine secretion of inflammatory factors, such as TNF-α, IL-6, IL-1β protein and MCP-1 factor, enhances Bcl2 levels, reduces bcl2-related X protein, apoptotic protease activator 1, caspase 3, and PARP activities, and decreases apoptosis levels. Breviscapine can also block CCl_4_-induced oxidative stress by reducing ROS production, improving antioxidants, and blocking mitogen-activated protein kinase pathways; by contrast, it can induce CCl_4_-induced acute liver injury and LPS induction by inhibiting inflammation and apoptosis of L02 cells, elicit a protective effect on apoptosis, and improve the histological changes and collagen deposition induced by CCl_4_ in mice (Liu Y. et al., 2018). According to histopathological analysis, DZHSI can reduce the levels of the liver enzymes aspartate and alanine aminotransferase, decrease MDA levels, and increase the SOD activity. Western blot and RT-q polymerase chain reaction have shown that breviscapine pretreatment can reduce the expression of mitochondrial fusion protein 2 (mfn2), caspase-3, and cytoplasmic cytochrome c protein. Therefore, breviscapine pretreatment may reduce lipid peroxidation, inhibit oxidative stress, and inhibit the protein and mRNA expression of Mfn2 to achieve a protective effect against liver ischemia–reperfusion injury (Lin et al., 2016; Bao et al., 2018).

### Breviscapine Pills (DZHSP)

#### Cardiovascular Diseases

Similarly, in pharmacological experiments, it was found that DZHSP mainly exerts anti-inflammatory effects by lowering blood lipids, changing hemodynamics, reducing interleukins, tumor necrosis factor and other inflammatory factors, increasing the activity of SOD in the body, and reducing caspase and other apoptotic factors, thereby exerting anti-oxidation, anti-apoptosis of cardiomyocytes, improve symptoms of cardiovascular disease (Shi et al., 2016; Wu et al., 2017). See the Table 4 for more research.

#### Liver Diseases

Give DZHSP intragastric treatment to rats with ischemia-reperfusion liver injury. The degeneration and necrosis of hepatocytes are reduced, ALT and AST are significantly reduced, and SOD activity is significantly increased (An et al., 2013). DZHSP improves the body’s elimination of free radicals through anti-oxidation and enhancement of SOD activity, and ability to protect liver function and reduce liver damage. For liver transplantation donor liver, DZHSP pretreatment can significantly inhibit inflammation-related factors and apoptosis-related pathways and protect microcirculation endothelial cells to reduce ischemia-reperfusion injury after liver transplantation in rats (Zhong et al., 2016). See the Table 4 for specific research.

#### Pulmonary Diseases

DZHSP reduces the pulmonary artery pressure in rats with chronic hypoxic pulmonary hypertension, inhibits the proliferation of pulmonary arterioles media smooth muscle cells and the production and accumulation of pulmonary artery wall collagen, reduces the activation and expression of PKC, and blocks various PKC signaling pathways thereby Inhibit chronic hypoxic pulmonary hypertension and pulmonary vascular remodeling. See the Table 4 for specific research.

#### Kidney Diseases

The animal model of kidney injury was treated with DZHSP and found that DZHSP inhibits reactive oxygen species (ROS) and kidney injury molecule-1 (KIM-1), reduces the expression of TNF-α, MCP-1, MIP-2 inflammatory factors, inhibits inflammatory cells chemotaxis and activation, reduce the level of selectin expression, inhibit oxidative stress, reduce lipid peroxidation and free radical damage, thereby reducing renal ischemia-reperfusion injury (Jia et al., 2011; Zhao et al., 2019). See the Table 4 for specific research.

#### Diabetes and Complications

It can also be found in diabetic nephropathy models that DZHSP can reduce renal fibrosis and tubular damage, improve the expression of fibrosis markers of diabetic nephropathy, reduce proteinuria and serum creatinine, and phosphorylate PKCβII/Akt/JNK1/2/p38 signaling pathway Inhibit renal fibrosis (Jiang et al., 2016). See the table for specific research. After diabetic retinopathy (DR) rats were treated with DZHSP intragastrically for 120 days, the lipid peroxide level in the retina was significantly reduced, and the superoxide dismutate activity was significantly increased, suggesting that DZHSP may enhance the antioxidant capacity and Inhibit retinal cell apoptosis to improve retinopathy (Han, 2009). See the Table 4 for specific research.

### Scutellarin

As the main active ingredient studied in DZXX preparations, scutellarin has multiple effects such as anti-oxidation, anti-free radical, anti-coagulation, anti-inflammatory, anti-apoptosis and anti-fibrosis. See Table 4 for the pharmacological studies.

Pharmacological experimental studies have shown that in the MCAO rat model, scutellarin inhibits the release of TNF-α, IL-1β and other inflammatory factors, reduces the expression of MMP-9, Caspase 3, increases the expression of anti-apoptotic factor Bcl-2, and prevents Extracellular matrix damage and blood-brain barrier destruction, inhibit cell inflammation and apoptosis, promote the recovery of nerve function, and improve cerebrovascular diseases (Wang L. Z. et al., 2006; Yang et al., 2019a; Chen et al., 2020).

Taking cognitive dysfunction rats as the research object, it was found that scutellarin can significantly improve their learning and memory decline. Further research found that scutellarin reduces the content of MDA in the hippocampus, increases the level of SOD, and reduces the inflammation Factors NF-κB, TNF-α, IL-6 expression, reduce the beta amyloid precursor protein (APP) in brain tissue, reduce the generation of free radicals, inhibit neuronal toxicity, reduce cell apoptosis and inflammation, thereby improving recognition Cognitive dysfunction (Guo et al., 2011a; Guo et al., 2011b; Guo and Guan, (2013); Wang Y. J. et al., 2018).

It has also been found in animals and cell models of myocardial ischemia that scutellarin mainly improves dyslipidemia, increases tPA levels against platelet aggregation, increases fibrinolytic activity, reduces the expression of MMPs, stabilizes heart plaques, and reduces the expression of inflammatory factors in cardiomyocytes Scutellarin can also reduce ROS and MDA content, increase SOD activity, increase cell viability and mitochondrial membrane potential, inhibit myocardial inflammation, apoptosis and oxidative stress, and reduce myocardial cell damage.

In an animal model of lipopolysaccharide-mediated liver cell injury, scutellarin significantly increases the activities of SOD, GSH-Px, and CAT in liver tissues, reduces the content of XOD, MDA, and NO, and resists lipid peroxidation, reduces the production of lipid peroxide, enhances the body’s ability to scavenging free radicals, expands blood vessels, improves liver hemoperfusion and protects the liver (Yang and Guo, (2010)). In addition, scutellarin can also reduce the content of TNF-α, IL-6, IFN-γ inflammatory factors to improve liver inflammatory response and reduce liver damage (Niu et al., 2015). And scutellarin was found to reduce the expression of apoptotic factor Stat3 in liver cancer cells, and inhibit the metastasis of liver cancer cells by inhibiting the STAT3/Girdin/Akt signaling pathway (Xu and Zhang, 2013).

Scutellarin can inhibit the proliferation, migration and invasion of renal cancer cells (ACHN, 786-O) in a dose-dependent manner *in vivo* and *in vitro*, and induce their apoptosis, and significantly reduce cyclin D1 (cyclin D1) and cyclin-dependent kinases (CDK1), Bcl-2, MMP-2, MMP-9 and other key protein expression, enhance the expression of Bax, cleaved Caspase-3 and p21, induce cancer cell apoptosis, and also increase PTEN by inhibiting the P13K/AKT/mTOR pathway, partially inhibit the proliferation and invasion of renal cell carcinoma (Deng et al., 2018). In addition, scutellarin can also reduce the levels of TNF-α and IL-6 in kidney tissue to exert anti-inflammatory effects, up-regulate the expression of nuclear factor red-like 2 related factor 2 (Nrf2), and increase heme oxygenase 1 (HO-1), regulate the Nrf2/HO-1 signaling pathway to play a role in lowering blood sugar and renal protection (Liu et al., 2019; Sun et al., 2019).

In diabetic animals and cell models, it was found that scutellarin can reduce the blood sugar level of diabetic rats by improving inflammation and anti-oxidation. In addition, scutellarin up-regulates the expression of Nrf2 and promotes the expression of HO-1, SOD, CAT in the kidney, which may inhibit the oxidative damage of the kidney through the Nrf2/HO-1 signaling pathway and improve diabetic nephropathy (Liu T. et al., 2018; Mei et al., 2019; Gao et al., 2020). Scutellarin can reduce the expression of NF-κB, TNF-α, ERK1/2, reduce retinal damage caused by the activation of microglia during the development of diabetic retinopathy, and can also inhibit VEGF and its downstream protein p-ERK, phosphorylate focal adhesions Kinase (p-FAK), phosphorylated tyrosine protein kinase (p-Src), inhibits the angiogenesis of diabetic retinopathy by down-regulating vascular endothelial growth factor/ERK/FAK/Src pathway signals, and improves microvascular dysfunction (Mei et al., 2019).

## Pharmacokinetics

Studies on the pharmacokinetics and absolute bioavailability of scutellarin in dogs have shown that after the intravenous administration of scutellarin, it is metabolized, is excreted rapidly, and has a short elimination half-life; its oral administration is almost not absorbed, and the absolute bioavailability is only 0.2–0.75% (Liu et al., 2002). The main reasons for the low oral bioavailability of scutellarin are low solubility in gastrointestinal fluid, poor membrane permeability, first-pass metabolism in the gastrointestinal tract, and efflux of transport proteins. The average bioavailabilities of 1,5-dicaffeoylquinic acid (1,5-DCQA) in dogs and rats are only 3.50 and 0.52%, respectively. The causes of low bioavailability of caffeic acid esters were intestinal metabolism, poor self-absorption and efflux of transporters (Xia, 2016).

After the oral and intravenous administration of breviscapine, the drug-time curve has a multipeak phenomenon. Further experiments should confirm whether the multi-peak phenomenon is related to the liver and intestinal circulation. The *in vivo* process of total caffeic acid esters in DZXX injection conforms to the two-compartment model of intravenous injection. At the same time, the drug–time curve shows a multipeak phenomenon. Because scutellarin in DZXX injection is also an effective ingredient for promoting blood circulation and removing blood stasis, whether the pharmacokinetic behavior of the two ingredients influences each other needs to be further explored (Zhang et al., 2005; Li et al., 2007b; Li et al., 2007c). Studies have shown that scutellarin is mainly absorbed in the intestinal tract via passive diffusion, and absorption is linearly related to concentration in the range of 50–400 μg/ml, and absorption is not affected by pH at pH 6.0–7.4. Ge Qinghua et al. found that intravenous administration of 90 mg or 1.8 g of scutellarin in beagle dogs is rapidly eliminated in the body after intravenous administration, whereas the absolute bioavailability of oral administration is only 0.40 ± 0.19% (Ding and Jiang, 2003; Ju et al., 2005). Rats are intragastrically given 80 mg/kg scutellarin after 4, 8, and 12 h after administration; the content of scutellarin in the kidney is the highest, followed by the heart, liver and brain. After scutellarin is injected into the blood, the half-life of the distribution phase is very short, i.e., 1.3 min in rabbits. Domestic dogs are only 7 min (Jiang et al., 2003). Rats are given the same amount of scutellarin aglycone and scutellarin via gavage. Scutellarin aglycone is easily absorbed through oral administration. The relative bioavailability of scutellarin aglycone is 301.8% compared with that of scutellarin (Che et al., 2006). In addition, the half-life (*t*_1/2_) and residence time (MRT) of caffeic acid in breviscapine injection in rats were significantly higher than that of caffeic acid monomer alone. Certain components in breviscapine injection are different from caffeic acid interacts, prolonging the time of caffeic acid in rats (Dai et al., 2013). Aspirin injection combined with DZXX injection in rat tail vein, T1/2β of coffee increased significantly, the clearance rate of CL decreased, and the area under the plasma concentration–time curve AUC (0–t) and the surface volume of distribution (Vd) increased, indicating that aspirin can slow down the metabolic process of caffeic acid in the body (Dai et al., 2014). After the intravenous injection of scutellarin liposomes in beagle dogs, the blood concentration is greatly increased, the pharmacokinetic properties of the scutellarin original drug are significantly improved, and it has a sustained release effect (Lv et al., 2006)..

In a PK-PD study, after the MCAO model of rats, a one-time intravenous bolus of DZXXI (5 ml/kg/day), from 5 min to 50 h after cerebral ischemia, can reduce cerebral infarction rate in MCAO rats. The onset of T is cerebral ischemia for 5 min, the duration of T is 48 h, T_max_ is 24 h, T1/2 is 21.84 h, and E_max_ is 11.71%. DZXXI reduces cerebral infarction rate in MCAO model rats with fast onset and long maintenance time. The reduction has the characteristics of quick onset and long maintenance time and provides a reference for specific drug screening, optimal dosing regimen, and clinical rational use of ischemic stroke. The peak of drug influence lags the peak of plasma concentration, and the effect of reducing the cerebral infarction rate is negatively correlated with the average blood concentration of the seven chemical components at 5–10 min of cerebral ischemia and positively correlated at 10 min–6 h; in addition, the time-quantity relationship of seven chemical components was negatively correlated. The pharmacokinetic values of the seven chemical components in Dengzhanhua injection were the highest 5 min after cerebral ischemia. The lowest detectable values of baicalin and isochlorogenic acid B appeared after 6 h of cerebral ischemia and could not be detected after 8 h. The lowest detectable value of 5-caffeinyl quinic acid and 4,5-bisphenol quinic acid appeared at the 3rd hour of cerebral ischemia and no longer visible at the 4th hour. The lowest detectable values of 4-caffeinylquinic acid, 3,5-bisphenol-quinic acid, and chlorogenic acid were observed 2 h after cerebral ischemia and no longer detected after 3 h (Liu G. et al., 2020).

## Toxicological Research and Safety Evaluation

From the records of traditional literature to the acute and subacute toxicity experiments of this product, DZXX has low toxicity and is a safe medicinal material. Subacute toxicity test shows that scutellarin (mainly containing scutellarin B and scutellarin A) has no effect on blood, liver and kidney functions, and no substantial changes in organs (Wang Z. et al., 2012).

The acute toxicity test of DZXX injection shows that the mice developed abnormal mental behavior and breathing, followed by behavioral disorders, convulsions, and death after a single intravenous or intraperitoneal injection of *E. breviscapus* injection. Female and male intravenous bolus injections of DZXX injection measured with the Bliss method had LD_50_ of 1,676.75 and 1,744.76 mg kg^−1^, respectively, and no significant difference were detected. Long-term toxicity test observe that the rats intraperitoneally injected with 480 mg kg^−1^ DZXX once a day for 2 months, its body weight increased slowly, and pathological examination found that some renal tubular epithelium in the cortex of renal tissue had mild turbid swelling. The weight gain of rats was slow, and pathological examination shows that some renal tubular epithelia in the inner cortex of renal tissues had mild turbid swelling, but 120 and 30 mg kg^−1^ dose groups did not cause drug damage and delayed toxicity after drug treatment was terminated. The results of the long-term toxicity test of DZXX injection in beagle dogs show that animals in the 160 mg kg^−1^ dose group of DZXX injection were continuously administered intravenously for 60 days, and drug damage and reactions occurred after drug withdrawal. Blood biochemical examination revealed that treatment for 30 days increases creatinine levels. Some animals in the 40 mg kg dose group also had drug reactions during the administration, and their blood biochemical examination indicated that the total protein content increased after administration. The dose of 10 mg kg had no significant effect on mental behavior, blood and urine biochemical examinations (Guo and Li, 2012).

A safety test study on breviscapine injection has shown that breviscapine was intravenously injected into the abdominal cavity of guinea pigs for three consecutive times for 14 and 21 days after the administration, and no allergic reaction was found. with the rabbit ear margin intravenous injection of DZXX injection 1 time/day for three consecutive days, no obvious irritation was observed at the injection site. At the same time, hemolytic test showed that breviscapine injection had no hemolysis and agglutination. The rabbits were injected intramuscularly with breviscapine injection. No obvious hyperemia, redness, and swelling were observed on the surface of the muscle. The rabbits had normal activities and no obvious abnormal reactions. After execution, the order of muscle irritation at the administration site was level 0; the rabbits were continuously injected with the drug in the ear vein once a day. After the administration for 5 days, no abnormal reaction was observed in the ear veins. The microscopic examination of the tissue section revealed that the endothelium of the ear veins was intact and smooth. No inflammatory reaction was found in the wall of the ear vein and no blood column formation in the lumen (Liang and Su, 2014).

The rats were continuously administered through the intraperitoneal injection of *E. breviscapus* for 90 days. The drug was discontinued every 7 days for 14 days. The main manifestations were fluctuations in blood routine indicators and prolonged PT and aPTT. The high-dose group of 20 crude drugs/g and the middle-dose group of 10 crude drugs/g in all female and male rats had lower HCT (*p* < 0.01 or *p* < 0.05) and decreased in a dose-dependent manner. WBC, YLM, and MID decreased (*p* < 0.01), but they were similar to the values after 90 days of administration, and no abnormality was detected in body weight and liver function, which was presumed to be the performance of the drug’s blood-activating and stasis-removing effect. Indicators can be restored after the drug administration was terminated. No obvious delayed toxicity was observed, and the safe dose range was 20 g/kg and below (Wu et al., 2007).

The acute toxicity test of compound Dengzhanhua dripping pills was performed on mice once a day and observed for 7 days. Within 7 days after the administration, the physiological conditions of the mice were normal, their weight increased, and no death occurred. In a long-term toxicity test, rats were intragastrically treated once per day at doses of 0.9, 0.45, and 0.225 g/kg for 10 weeks of continuous administration and withdrawal for 2 weeks, the body weight, blood biochemical examination, organ coefficient, organ tissue structure, and other aspects of rats in each group were normal, and no obvious specific pathological changes related to drug toxicity were found (Wan et al., 2003).

The oral toxicity of DZXX extract is relatively small. When a mouse is given 80 g kg^−1^ through gavage once, no death was observed within 3 days; LD_50_ (ip) = 13.14 ± 5.43 was calculated with the simplified probability unit method (g kg^−1^), LD_50_ (iv) = (10.02 ± 1.55) g kg^−1^ (Wang and Wang, 1985; Liang and Su, 2014).

The ADRs of DZXX-related preparations mainly include allergic reactions, nervous system reactions, digestive system reactions, cardiovascular system reactions, respiratory system reactions, and blood system reactions. ADR symptoms include rash, chills, fever, and shortness of breath, palpitation, headache, lower extremity edema, elevated blood pressure, and abdominal pain. The top 10 ADR symptoms are pruritus, rash, dizziness, chills, palpitation, headache, fever, suffocation, nausea, and flush. The most common systems and organs affected by ADRs are damage to the skin and its accessory organs. Men have more adverse reactions than women. The most severe ADR occurs in elderly patients aged 60 years and older (Li Y. et al., 2015).

## Conclusion

According to the *China Cardiovascular Disease Report 2018*, the current number of cases of cardiovascular diseases in China is estimated to be 290 million, including stroke 13 million, coronary heart disease 11 million, pulmonary heart disease 5 million, heart failure 4.5 million, rheumatic heart disease 2.5 million, congenital heart disease 2 million, hypertension 245 million. Cardiovascular and cerebrovascular diseases are currently the number one cause of death among Chinese residents. The incidence increases and tends to affect younger individuals. The mortality rate in rural areas is higher than that in urban areas. In 2016, the death rate of cardiovascular and cerebrovascular diseases in rural areas was 309.33/100,000. Of these cases, the death rate of heart disease was 151.18/100,000. In urban areas, the death rate of cardiovascular and cerebrovascular diseases was 265.11/100,000. Of these cases, the death rate of heart disease was 138.70/100,000. Hypertension, hyperlipidemia, diabetes, obesity, and elevated blood uric acid are the main risk factors of cardiovascular and cerebrovascular diseases. With the increase in the number of patients with cardiovascular and cerebrovascular diseases, the demand for drugs increases yearly. The sales of cardiovascular drugs in China are second only to anticold and gastrointestinal drugs, ranking third. The amount of sales of cardiocerebral vascular drugs in China has shown an increasing trend, that is, it increased from 60.93 billion yuan in 2013 to 83.44 billion yuan in 2017, with a compound annual growth rate of 8.2%. Therefore, the pharmaceutical market’s demand for DZXX raw materials and biological drugs has also increased (Zhou, 2006; Hu et al., 2019). According to reports, in 2019, the sales revenue of traditional Chinese medicine preparations composed of DZXX as a raw material was about 3 billion yuan.

At present, the preparation of marketed DZXX varies. For example, breviscapine granules are extracted with alcohol, and DZXX injection is extracted with water. The chemical compositions of similar products from different manufacturers differ, and different batches of the same pharmaceutical company also have significant differences. Such differences are unreasonable. In addition, chemical fingerprint analysis via HPLC-DAD and other techniques has revealed that the chemical composition spectrum is also significantly different. Although DZXX and breviscapine injections differ in terms of preparation processes and chemical compositions, their clinical functions and indications are the same. It can be inferred that scutellarin is one of the common substance base, but it also suggests that there are still a large number of invalid components in different preparations in theory, and it is necessary to further explore the basis of pharmacodynamic substances to provide guarantee for the improvement of prescription and technology. Therefore, for the different preparations of *E. breviscapus* included in the national drug standards, the rationality of existing traditional Chinese medicine preparations should be studied in terms of their curative effect or efficacy. Improvement plans should be carried out on the premise of focusing on the material basis of drug effects. The preparation process and quality standards of related preparations should be standardized, some of the preparations should be eliminated on the basis of effectiveness, and the preparations of different processes should be compared and examined to find a more comprehensive material basis for using original medicinal materials. Based on HPLC and other techniques, the quality control methods of fingerprinting and determination of pharmacodynamic components from medicinal materials from medicinal materials, intermediate raw materials to preparations, and qualitative and quantitative studies of flavonoids, caffeoyl and other chemical components in DZXX related preparations were carried out to ensure the homogeneity, safety and stability of product quality, and to further improve the quality control level of breviscapine asarum and lay the foundation for the entry of breviscapine asarum into the international market (Wang, 2009; Zhou, 2006).

The bioavailability of oral preparations of DZXX medicinal components is low. Their injections have a short half-life and are rapidly eliminated from the body. They are also associated with poor patient compliance and inconvenient use. Therefore, the development of a drug delivery system with simple preparation process, high drug loading, improved oral absorption, and improved bioavailability of baicalin and total caffeic acid ester as main pharmacodynamic components of breviscapine is the main breakthrough point in future work, and is also the key link to give full play to the advantages of breviscapine in the treatment of cardiovascular and cerebrovascular diseases. Studies on new formulations of *E. breviscapus* have focused on the same aspects of DZXX preparations. Most of them have explored the bioavailability of flavonoids but have rarely investigated the simultaneous improvement of the main effective components of breviscapine. Coffee acyl components occupy a large proportion of DZXX, but the present research on light lamps mainly focuses on flavonoids, coffee acyl components also have good activity. Research has shown that 3,5-di-O-caffeoyl quinic acid can increase serum SOD, GSH-Px, NOS activities and reduce MDA content in MCAO rats to increase BBB permeability, and improve cerebral ischemia. Studies on the bioavailability of baicalin and total caffeic acid esters, the mechanism of action, pharmacodynamics, and structure-activity relationships of the active ingredients of DZXX should be performed in detail (Sheng et al., 2016). Furthermore, research on new formulations is insufficient at cellular and molecular levels, at the same time; there are still many deficiencies in the study of animal and human for pharmacokinetics. Therefore, studies on the drug release characteristics, transport in the body, absorption kinetics, bioavailability, and efficacy of *E. breviscapus* should be conducted (Xia, 2016).

DZXX has complex chemical components and extensive pharmacological effects. It has the characteristics of multiple components, multiple targets, and overall regulation when it exerts its drug effects. Studies on the effect of DZXX on some metabolites in rats with ischemic brain injury have verified and explained the traditional mechanism, but studies have yet to discover new biomarkers and mechanism of action. Recent studies were based on the BBB, and they involved the use of the ROS/RNS-MMP-TJ signaling pathway as the entry point to explore the molecular mechanism of DZXX injection that protects the BBB damage caused by cerebral ischemia in rats. However, mechanisms and pathways in diseases treated with DZXX remain unclear. Therefore, methods such as network pharmacology and HPLC should be applied to study the distribution of the active ingredients of DZXX-related preparations, therapeutic targets, and signal pathways, to determine and clarify their chemical composition, to investigate their mechanism of action and regulation, and to provide a scientific theoretical basis for clinically applying DZXX-related preparations.
